# Dynamics & Spectroscopy with Neutrons—Recent Developments & Emerging Opportunities

**DOI:** 10.3390/polym13091440

**Published:** 2021-04-29

**Authors:** Kacper Drużbicki, Mattia Gaboardi, Felix Fernandez-Alonso

**Affiliations:** 1 Materials Physics Center, CSIC-UPV/EHU, Paseo Manuel de Lardizabal 5, 20018 Donostia-San Sebastian, Spain; kacper.druzbicki@ehu.eus; 2 Polish Academy of Sciences, Center of Molecular and Macromolecular Studies, Sienkiewicza 112, 90-363 Lodz, Poland; 3 Elettra—Sincrotrone Trieste S.C.p.A., S.S. 14 km 163.5 in Area Science Park, 34149 Trieste, Italy; mattia.gaboardi@elettra.eu; 4 Donostia International Physics Center (DIPC), Paseo Manuel de Lardizabal 4, 20018 Donostia-San Sebastian, Spain; 5 Department of Physics and Astronomy, University College London, Gower Street, London WC1E 6BT, UK; 6 IKERBASQUE, Basque Foundation for Science, Plaza Euskadi 5, 48009 Bilbao, Spain

**Keywords:** neutron spectroscopy, computational materials modeling, soft matter, plastic crystals, polymers, supramolecular frameworks, nuclear quantum effects, water, ice, sustainable materials

## Abstract

This work provides an up-to-date overview of recent developments in neutron spectroscopic techniques and associated computational tools to interrogate the structural properties and dynamical behavior of complex and disordered materials, with a focus on those of a soft and polymeric nature. These have and continue to pave the way for new scientific opportunities simply thought unthinkable not so long ago, and have particularly benefited from advances in high-resolution, broadband techniques spanning energy transfers from the meV to the eV. Topical areas include the identification and robust assignment of low-energy modes underpinning functionality in soft solids and supramolecular frameworks, or the quantification in the laboratory of hitherto unexplored nuclear quantum effects dictating thermodynamic properties. In addition to novel classes of materials, we also discuss recent discoveries around water and its phase diagram, which continue to surprise us. All throughout, emphasis is placed on linking these ongoing and exciting experimental and computational developments to specific scientific questions in the context of the discovery of new materials for sustainable technologies.

## 1. Overview

The use of the neutron as an exquisite probe of the structure and dynamics of condensed matter is not only well-established but also continues to evolve in exciting directions. Currently under construction in Lund (Sweden), the European Spallation Source (ESS) seeks to offer order-of-magnitude gains in capability relative to the state-of-the-art, as presented in the recent overview of its forthcoming instrument suite by Andersen et al. [1]. Equally, recent advances towards the design and construction of increasingly more compact neutron sources across the globe are pushing the boundaries of the discipline into entirely uncharted territory [2,3,4,5,6,7,8,9], as well as it is providing fertile new ground and a plethora of fresh opportunities for further developments, much in the same way as it happened when X-ray sources entered the laboratory some decades ago [10].

Progress in neutron science over the past few decades and up until a few years ago has been extensively reviewed in a series of three recent monographs [11,12,13]. Among the wide range of techniques available at present, Neutron Spin-Echo (NSE) and QuasiElastic Neutron Scattering (QENS) have been the most-commonly used to explore the dynamics of soft matter [14,15,16,17,18,19,20,21]. Recent highlights in QENS include the implementation of neutron polarization analysis on a wide-angle time-of-flight spectrometer at a pulsed neutron source, allowing a clean separation of coherent and incoherent contributions with sub-meV resolution and over a wide momentum-transfer (*Q*) range [22]. In contrast, the widespread use of Inelastic Neutron Scattering (INS) can still be regarded in its infancy compared to the above [23,24,25], although its potential to explore a number of important and relevant phenomena has been recognized for some time now, including: crystallinity [26,27,28]; methyl [29,30] and short-chain dynamics [31]; the vibrational dynamics of polymer glasses [18,32,33,34,35,36]; or even (although more rarely) polymer thin-films [37,38,39]. For the purposes of this review, INS would refer to the study of well-defined modes at energy transfers (*E*) ranging from a few meV all the way up to ca. 500 meV, the highest energy characterizing the vibration of the lightest molecule, H${}_{2}$.

Beyond the aforementioned upper bound for INS, the use of epithermal (electron-volt) neutrons leads to Neutron Compton Scattering (NCS), also referred to as Deep Inelastic Neutron Scattering (DINS). NCS gives access to mass-resolved nuclear mean kinetic energies and the underlying momentum distributions, of relevance to the study of quantum effects in condensed-matter systems [40]. Traditionally, NCS has been particularly useful in the study of hydrogenous matter [41], yet at the same time the technique continues to be extended to heavier elements under the wider umbrella of MAss-resolved Neutron SpEctroscopic (MANSE) techniques [42,43,44,45,46,47,48,49]. Neutron spectrometers in a so-called inverted-geometry configuration at a pulsed source represent the most natural way to implement NCS and MANSE, as exemplifed by the current science programme on the VESUVIO spectrometer (ISIS, UK) [50,51,52,53,54,55,56,57,58,59], although it is also to be noted that other approaches have been attempted in the past [60,61,62].

**Figure 1:**
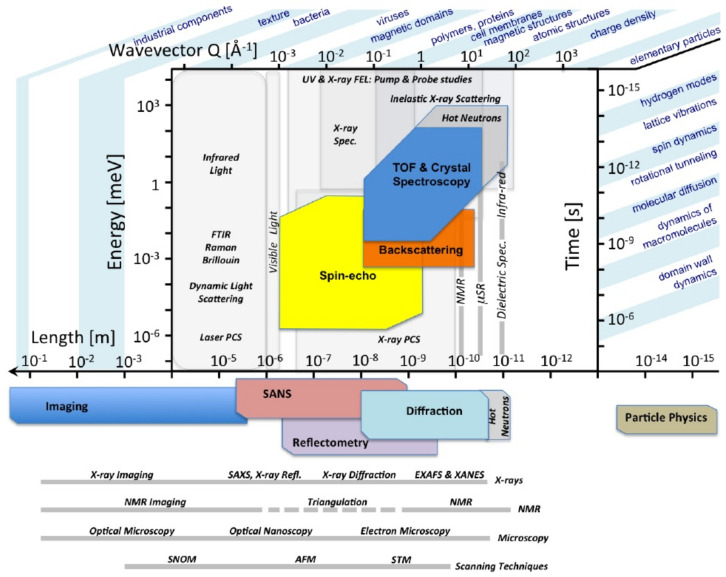
Regions of *Q* and *E* simultaneously accessible to neutrons, and qualitative comparison with other experimental probes. For ease of comparison, associated length and time scales are given by the bottom and right axes, respectively. Neutron spectroscopic techniques include the use of epithermal (hot neutrons), thermal (time-of-flight and crystal spectroscopy), and cold/ultracold wavelengths (backscattering and NSE). Blue text entries around the main figure illustrate areas of scientific and technological application. Reprinted with permission from Ref. [63].

The primary purpose of this contribution is to highlight recent advances and applications of INS and NCS in the context of soft matter. For the benefit of both novice and expert, emphasis is placed on the new tools and methodologies that have become available to interrogate the dynamical behavior of a growing range of complex materials of relevance to sustainable applications, particularly those of a soft and polymeric nature. In doing so, we also seek to bring to the fore the unrivalled ability of neutron spectroscopic techniques to probe dynamical phenomena over a wide range (decades) in timescales, from slow stochastic and relaxation phenomena in disordered media to the ultimate effects of quantum mechanics on properties and function (see Figure 1). To this end, we have chosen a limited number of areas of research that serve to illustrate the most salient points and take-home messages that we would like to convey to the reader, particularly to those who have shied away until now from the use of these superb tools of scientific inquiry.

## 2. Soft Media for Photovoltaics & Photonics

A class of solids currently dubbed Organometal Halide Perovskites (hereafter OHPs) were nothing more than a scientific curiosity not so long ago, practically unknown to most but a handful of materials scientists interested in their rather unusual properties as soft ‘plastic’ crystals [64]. This situation has changed dramatically since 2009 [65], following the use of the OHP methylammonium lead iodide (molecular formula CH${}_{3}$NH${}_{3}$PbI${}_{3}$, hereafter denoted MAPbI${}_{3}$) as a sensitizer in solar cells [66,67]. A decade on, top-performing OHP-based devices have attained record conversion efficiencies of solar light into electricity of 20–25%, a figure well above the (rather-shy) value of 3.8% of a decade ago, and comparable to far-more-costly silicon-based technologies [68]. Notwithstanding this progress, critical issues remain, primarily associated with their physico-chemical stability and their propensity to degrade [69,70]. Circumventing these difficulties requires a robust and coherent understanding of OHPs at the atomic and molecular levels, and this task has and continues to be a challenge to both experiment and theory. This is where recent developments in neutron spectroscopy come into the picture.

**Figure 2:**
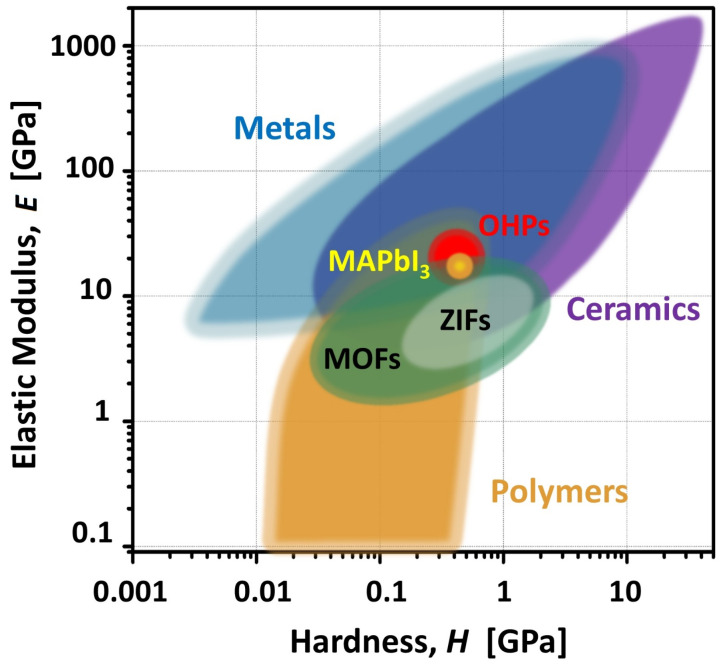
Color map of the elastic properties of several classes of materials around ambient conditions, including those discussed in the present and subsequent sections of this work. Please note the logarithmic scales of both vertical and horizontal axes. Adapted from Ref. [74] with permission from The Royal Society of Chemistry.

Chemically speaking, OHPs arise from the insertion of an organic cation into an inorganic perovskite lattice, replacing the more traditional metal ion. Such a substitution gives rise to an incredibly rich and complex structural diversity, as well as to new properties associated with the additional degrees of freedom introduced by the organic cation. Among the OHPs studied to date, the ones containing methylammonium (MA^+^) and formamidinium (FA^+^) moieties confined in iodo- and bromoplumbate frameworks are the most archetypal ones, and as such they will be used as a guide for further discussion below. This hybrid organic-inorganic chemical composition also translates into an unusual softness and flexibility, which gives rise to a rich polymorphism [71]. In terms of elastic properties, Figure 2 serves to illustrate where OHPs sit relative to other classes of materials [72,73], essentially at the cross-roads between the ‘*soft*’ and ‘*hard*’ worlds, and comparable to typical polymer or macromolecular systems of an organic nature.

**Figure 3:**
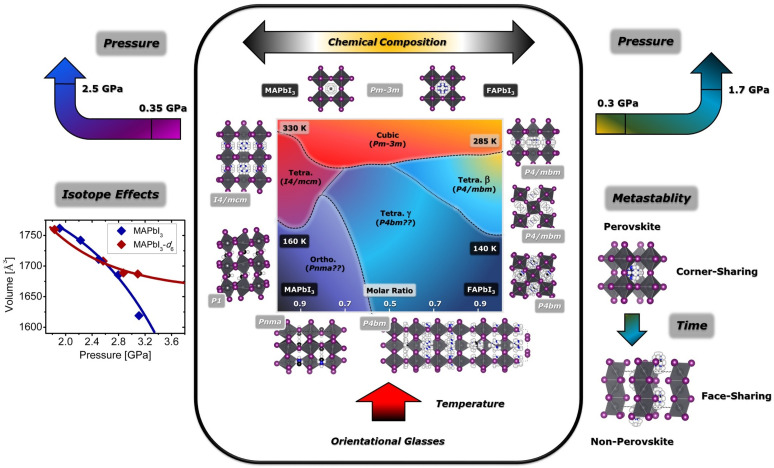
Phase diagram of ${\mathrm{MA}}_{1-x}{\mathrm{FA}}_{x}{\mathrm{PbI}}_{3}$ solid solutions over the temperature range 10–365 K at ambient pressure. Pure MAPbI${}_{3}$(FAPbI${}_{3}$) corrresponds to the left (right) edges of the diagram. For further details, see the main text. Adapted with permission from Refs. [75,81]. Copyright (2021) American Chemical Society.

The structural complexity of the iodoplumbates is further illustrated in the compositional phase diagram of ${\mathrm{MA}}_{1-x}{\mathrm{FA}}_{x}{\mathrm{PbI}}_{3}$ solid solutions (Figure 3) [75]. In this Figure, the arrows next to ‘*Pressure*’ indicate additional phase transitions as a function of this parameter for the parent compound [76]. Other phenomena illustrated in the figure include: strong isotope effects as a function of pressure (left) [77]; thermodynamic metastability leading to disproportionation into non-perovskite phases with poor photophysical response (right) [78]; or the emergence of orientational-glassy phases as a result of the dynamical arrest of the organic moiety (bottom) [79,80]. In addition, both MAPbI_3_ and FAPbI_3_ reveal a very complex polymorphism, closely linked to the progressive ‘*melting*’ of the organic cations within the solid. At ambient pressure, MAPbI_3_ shows three different crystal structures as a function of temperature: a low-temperature orthorhombic phase for T < 160 K; a tetragonal phase for 160 K < T < 330 K; and a high-temperature cubic phase for T > 330 K. For FAPbI_3_, the thermodynamically stable phase at ambient conditions is a non-perovskite hexagonal phase (yellow δ-polymorph). However, a metastable cubic perovskite phase (black α-phase) can be stabilized at RT for hours to weeks via heating above the hexagonal-to-cubic phase transition at 410 K. Upon cooling from the cubic phase, a tetragonal phase (β-phase) is formed below 285 K, and yet another structure is found below 140 K (γ-phase).

A substantial part of our current understanding of the structural properties of these materials stems from Neutron Diffraction (ND) [82,83,84,85,86,87,88,89,90,91,92,93,94]. Most importantly, the use of neutron Laue diffractometers, operating both at pulsed (e.g., SXD at ISIS, UK [95]; TOPAZ at SNS [96], US; SENJU at J-PARC, JP [97]) and continuous neutron sources (e.g., KOALA at ANSTO, AU [98]; FALCON at BER-2, DE [99]—currently transferred to the spallation source SINQ, CH) provide the means to precisely explore OHP single-crystal samples from a crystallographic perspective. Notwithstanding these efforts, the structure of both MAPbI_3_ and FAPbI_3_ remains elusive to a great extent, progress being hindered by the presence of both static and dynamical disorder, as well as by the formation of domain structures of multiple origins [75], or the propensity of these crystals to twin [100]. As a result, the assignment of the space group of each perovskite phase of both MAPbI_3_ and FAPbI_3_ discussed above has been debated from the outset [75]. The strong tendency of MAPbX_3_ to twinning, for example, arises from the ferroelastic relations between the high- and low-temperature phases, where the samples first nucleate and then crystallize, respectively [100]. This phenomenon has been recently explored by Breternitz et al. using single-crystal ND on the state-of-the-art instrument FALCON [94]. Similarly, the high-resolution total-scattering ND experiments on powder samples of FAPbI_3_ have brought to the fore the importance of partial disorder and the formation of an orientational glass to account for the metastability of the perovskite phases in this material. Extensive studies of the structural properties of FAPbI_3_ at ambient pressure can be found in Refs. [87,91].

Pressure studies of the structure of OHPs have been primarily carried out using synchrotron X-rays, with some ongoing debates on the space-group assignment of the high-pressure phases [76,100,101,102,103,104]. Parallel studies using neutrons continue to be quire sparse, largely limited by the large incoherent cross section from hydrogen and the need for per-deuterated specimens. Structural changes upon compression in (MA-*d*_6_)PbBr_3_ have been studied with powder ND up to *ca.* 2.8 GPa by Swainson and co-authors [105], yet even a fundamental understanding of the structural properties of this material at ambient conditions remains elusive [106]. Isotope effects have also been largely ignored in most high-pressure OHP research. In a recent paper, Kong and co-workers [107] used both high-pressure synchrotron and neutron diffraction (PLANET beamline, J-PARC, JP) and found that H/D substitution plays a critical role in suppressing lattice disorder in MAPbI_3_. Furthermore, per-deuteration gives rise to a large enhancement of light emission and structural robustness, acompanied by a slower degradation of photovoltaic performance.

**Figure 4:**
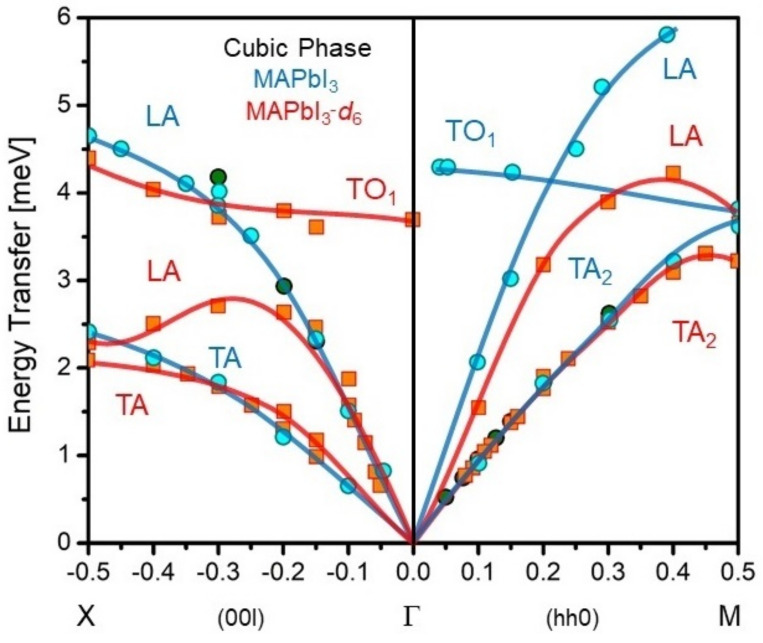
Phonon dispersion relations in the cubic phase of MAPbI_3_, measured on both perdeuterated and hydrogenous single-crystals using IXS (cyan circles [123]) and INS-TAS (orange squares [109] and green circles [113], respectively). Wave vectors on the abscissa are given in reduced lattice units. Longitudinal and Transverse Acoustic (LA and TA, respectively) branches are presented along with the lowest-energy Transverse Optical (TO) mode. Adapted with permission from Ref. [109].

Beyond structural studies, work to date on INS has been briefly reviewed by Mozur et al. [108]. These activities can be conveniently subdivided into coherent and incoherent INS experiments, the latter focused on the hydrogenous organic cations. The coherent phonon response brings information on the collective dynamics of the OHP framework, which can be effectively explored by neutron scattering. A number of neutron scattering instruments have been used so far for the study of OHPs. These instruments can be roughly classified as triple-axis spectrometers (TAS), operating at reactor sources (e.g., HB3, HFIR, ORNL, US [109]; BT4 and SPINS at NIST [110,111,112], US; IN12 and IN22 at ILL, FR. [112,113]; 4F1, 4F2 and 1T at LLB, FR [113,114,115,116]); and the time-of-flight (TOF) instruments available at spallation (e.g., MAPS and MARI at ISIS, UK [117,118,119]; 4SEASONS and AMATERAS at J-PARC, JP [120,121]; or ARCS at SNS, ORNL, US [120]) and reactor sources (IN4 and IN5 at ILL, FR [116,117,122]). However, as with ND, the incoherent scattering from hydrogen masks and obscures the coherent response. As a result, there have also been several reports on the successful application of Inelastic X-ray Scattering (IXS) to elucidate the collective dynamics of hydrogenous samples [88,123,124]. Overall, these studies of coherent lattice dynamics continue to provide essential information on the mechanical properties of the soft inorganic frameworks (mediated by acoustic phonons). In addition, they have facilitated a better understanding of charge-transport properties since the frontier electronic states of OHPs couple primarily to low-energy optical phonons [108]. The complementarity between inelastic neutron and X-ray scattering is illustrated in Figure 4a, showing phonon-dispersion relations for both per-deuterated and hydrogenous single crystals of MAPbI_3_ by means of INS-TAS and IXS, respectively. As clearly shown in these data, H/D substitution can lead to substantial shifts of the low-energy modes and the resulting elastic properties, thus serving as a timely cautionary note in future studies.

The first INS experiments on OHPs were reported by Swainson and co-workers, dealing with the case of MAPbBr_3_ [117]. This work identified the mode softening associated with rich polymorphic transitions in this system. Soon after, Létoublon et al. presented an exhaustive analysis of the low-frequency dynamics in MAPbBr_3_ with INS-TAS experiments combined with Raman and Brillouin scattering, and ultrasound measurements [115]. This line of research has been continued by the authors, providing the most comprehensive analysis of the mechanical properties of both MAPbX_3_ and FAPbX_3_ published to date [113]. INS has the advantage of providing microscopic information on the elastic properties of OHPs, since the acoustic phonons possess wavelengths which are considerably shorter than crystal dimensions. Moreover, it allows measurements in the THz range, where relaxation effects are negligible [125]. These works have built a picture of OHPs as materials with an exceptionally low shear elastic constant *C_44_*. For instance, the *C_44_* constants reported for FAPbBr_3_ and FAPbI_3_ by Ferreira et al. amount to 3.1 ± 0.1 GPa and 2.7 ± 0.3 GPa, respectively [113]. These values are comparable to 2.1 ± 0.3 GPa in crystalline polyethylene, measured with INS-TAS by Heyer et al. [125]. Furthermore, an analysis of the elastic properties of the cubic perovskite phase of FAPbI_3_ provides important clues on its metastability. The softening of the shear modulus with temperature also confirms the aforementioned ferroelastic relations in MAPbX_3_ perovskites, a pivotal finding to understand their strong tendency to exhibit twinning [100]. Twinning has been also evidenced by Li et al. in high-resolution experiments on a hydrogenous MAPbI_3_ single-crystal, performed with the chopper TOF spectrometer AMATERAS [121]. These experiments combined with extensive QENS and MD analysis of the underlying cation dynamics, allowed a clean distinction between the individual contributions from acoustic and optical phonons to the (exceedingly low) thermal conductivities of MAPbX_3_ perovskites. The authors proposed that the nanoscale mean-free-paths of acoustic modes together with lower velocities are responsible for such a small thermal conductivity and suggested a marginal influence of optical phonons on heat-transport properties [121]. These conclusions have been corroborated and explored further by Gold-Parker et al. [110] with INS-TAS experiments on per-deuterated MAPbI_3_, providing high-precision momentum-resolved measurements of acoustic phonon lifetimes, and demonstrating that acoustic phonons are unable to dissipate heat efficiently. These results highlight the importance of the so-called ‘phonon bottleneck’ effect, a phenomenon that opens the possibility of using OHPs in hot-carrier solar cell devices. In these circumstances, the loss of photogenerated charge carriers can be mitigated, thereby boosting device efficiency. Normally, carrier thermalization and cooling mechanisms rely on the coupling to optical phonons which, however, must further decay into acoustic ones and then dissipate. However, in some cases these phonons are produced at very high density so that they cannot decay away fast enough, allowing to scatter back and reheat the carrier ensemble. This phenomenon was examined in the aforementioned paper by Gold-Parker et al. [110], showing a pronounced inefficiency of acoustic-phonon-mediated heat-transport mechanisms in the MAPbI_3_ lattice. Yang et al. have explained the ‘*phonon bottleneck*’ effect in OHPs by acoustic-phonon up-conversion, which leads to a LO phonon emission rate ten times slower in FAPbI_3_ compared to the all-inorganic counterpart CsPbI_3_ [126]. The most-recent research on MAPbI_3_ further addresses the isotope effect revealed earlier by Kong et al. [77]. Manley et al. [109] have extended their previous INS-TAS experiments on a per-deuterated MAPbI_3_ [110,113] (see Figure 4a), revealing that isotope substitution in MA^+^ causes a large (20–50%) softening of the longitudinal acoustic (LA) phonons near zone boundaries, reduces thermal conductivity by nearly 50%, and slows carrier relaxation kinetics. These significant effects serve to emphasize once more out the importance of a coupling of LA phonons to the librational modes of the methylammonium molecule, which are strongly mass-dependent [109].

INS-TAS in combination with INS-TOF experiments also bring to the fore the role of optical phonons in the photophysics of these materials, as discussed in Refs. [109,127,128,129,130]. As noted by Mozur et al., optical phonons are responsible for over half of the polarizability in the dielectric response of MAPbI_3_ [108]. As deduced from the characteristic line shape broadening of the photoluminescence (PL), the main mechanism limiting the mobility of free carriers and excitons in OHPs is via the Fröhlich interaction between charge carriers and Longitudinal Optical (LO) phonon modes, which is, hence, the most important form of electron-phonon coupling in OHPs [108,116]. This phonon scattering mechanism is believed to be the key fundamental factor in establishing the intrinsic limit of charge carrier mobility [116]. State-of-the art INS experiments on optical phonons across the entire frequency range and over a wide *Q*-range have been recently presented and discussed by Ferreira et al. [116] (MAPX_3_ and FAPX_3_) and Zhang et al. [120] (MAPI_3_). Ferreira et al. have established the dispersionless character of optical phonons, overlapping with the upper part of the acoustic branches. The authors also highlight a significant anharmonic behavior, manifested as phonon overdamping at temperatures well below ambient. These results raise some question marks on commonly accepted ways of describing charge-carrier mobilities based on a quasi-particle picture for low-energy optical lattice modes [116]. Phonon damping was also examined by Zhang et al., probing the spatial and temporal coherence of the optical modes [120]. Moreover, the above phenomena do not appear to be restricted to OHPs. The very recent and elegant work by Lanigan-Atkins et al. [131] on CsPbBr_3_ show unambiguous signatures of liquid-like damping of Br-dominated phonons of a two-dimensional nature directly impacting the electronic gap-edge states.

Beyond the above, INS studies focusing on the organic moiety by exploiting the incoherent scattering from protons address another set of fundamental questions on the nature and coupling of cation and framework motions, of direct relevance to electron-phonon coupling and the overall photophysics of these materials (see, e.g., the discussion presented in Ref. [108]). The organic cation is known to accelerate the formation of large polarons that interact with both excitons and charge carriers, thereby extending the lifetime of the latter [108,132]. In addition, they participate in dynamical processes that reduce phonon lifetimes in these systems through a ‘*rattling*’ effect [133], which further reduces the macroscopic thermal conductivity. There is also mounting evidence on mode-specific phonon-bandgap coupling in OHPs [134], well beyond the seminal works on organic-rotor dynamics in the QENS domain [121,135]. The above-discussed papers have used both INS-TAS and direct-geometry INS-TOF instruments, with a focus on the low-frequency regime. A great advantage of TAS is their flexibility, allowing access to basically any *Q*-point, yet it can suffer from limited resolution and count rate. On the other hand, direct-geometry chopper TOF instruments offer simultaneous access across a wide range of both energy-transfers and scattering vectors, having large detector areas and so offering very-high sensitivity. On the other hand, for a direct-geometry instrument there is a significant resolution broadening, and the best resolution is only obtained when the energy of a given phonon is close to the incident energy selected by a mechanical chopper. Therefore, it is not possible to collect a high-resolution spectrum over the entire spectral range in one go, and merging different spectral regions is difficult because of the varying resolution functions [136]. This can become a serious limitation in molecular systems, where vibrational modes are typically spread out over a wide spectral range, including the celebrated ´*fingerprint*´ region. Indirect-geometry neutron spectrometers offer a means of circumventing these issues. Optimized to use short neutron pulses, the prime example is TOSCA, operating at ISIS, UK [137,138,139,140,141]. The key difference between TOSCA and direct-geometry instruments is the fact that it operates at specific and narrow energy-momentum-transfer kinematic trajectories optimized for the study of hydrogenous systems, thus providing a high and nearly constant relative resolution over a broad energy-transfer range. TOSCA has been subjected to significant upgrades over the last decade [137,138,139,140,141], which are still in progress and will result in even a higher detected flux. Capitalizing from this success, two broadband instruments of the same geometry have been developed at pulsed sources: VISION (SNS, ORNL, US [142]; *in operation*) and VESPA (ESS, SE [1]; *under construction*). LAGRANGE (ILL, FR) also shares the same layout to attain a high resolution, yet the steady-state nature of the source requires that the incident energy needs to be scanned [143]. Contrary to TAS or direct-geometry TOF spectrometers, this kind of instrumentation is characterized by a very high efficiency, allowing to study small samples within experimental time-scales comparable to standard optical vibrational spectroscopy techniques, i.e., infrared and Raman. These merits open the path for, e.g., in-situ and *operando* experiments in catalysis, or the use of high-pressure and gas-handling systems, opening new and exciting opportunities for chemistry and materials science. A limitation that needs to be kept in mind is the lack of access to small *Q*s at high *E*s, which results in a damping of the intensity of fundamental phonon excitations with energy transfer, accompanied by a concomitant increase of the intensity of overtones. Notwithstanding this limitation, this review amply demonstrates the versatility and power of this novel instrumentation in the study of molecular systems, where a high spectral resolution across a broad energy-transfer range becomes the key figure of merit.

**Figure 5:**
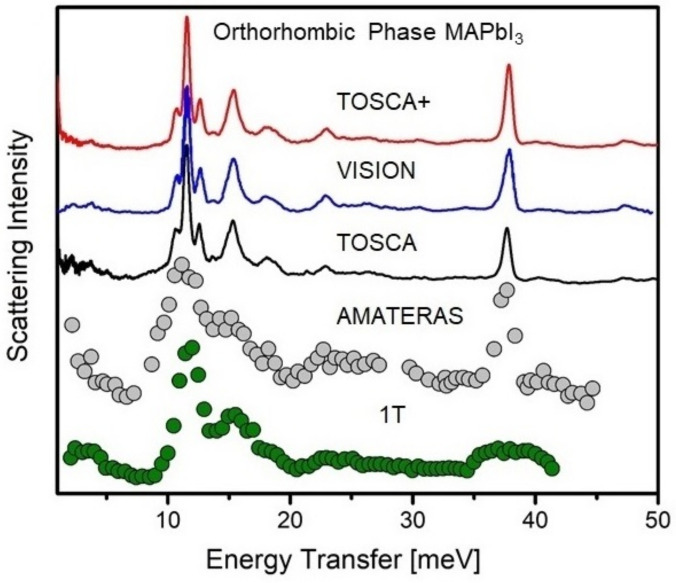
INS spectra of hydrogenous MAPbI_3_ in the orthorhombic phase at low temperatures (T = 5–20 K). These spectra were recorded on five different instruments: 1T (TAS; single-crystal [116]); AMATERAS (TOF; *E_i_* = 54 meV; single-crystal [121]; TOSCA [144], VISION [109], TOSCA+ [75] (indirect-geometry; powders). Adapted with permission from Refs. [75,109,116,121,144]. Copyright (2021) American Chemical Society.

Figure 5 serves to illustrate the above, with an example of the orthorhombic phase of MAPbI${}_{3}$ studied at cryogenic conditions using indirect-geometry (TOSCA and VISION), TAS (1T) [116], and chopper-TOF (AMATERAS, *E_i_* = 54 meV) [121] instruments. The spectral range has been limited to 50 meV, as, in this case, this range is key to understand the librational dynamics and the local structure in the vicinity of the organic cation. The first high-resolution INS studies of MAPbI${}_{3}$ over a wide spectral range were reported by Drużbicki et al. [144] using the previous incarnation of TOSCA (see the black curve in Figure 5). More recently, Manley et al. have reported the INS spectrum recorded on VISION [109] (blue curve in the figure), with a considerably better signal-to-noise ratio. These latter data are of comparable quality to those of a more recent study by Drużbicki et al. [75], obtained on an upgraded TOSCA, hereafter TOSCA+ [138,139,140] (red curve). While both VISION and TOSCA+ cannot offer the resolution at low-frequencies accessible with TAS or chopper-TOF instruments, recent developments on the state-of-the-art instrument OSIRIS, operating also at ISIS, provide unprecedented opportunities for THz-neutron spectroscopy [145].

The work of Drużbicki et al. [144] on the cation dynamics in MAPbI${}_{3}$ was heavily underpinned by the use of computational materials modeling to interpret the INS data, which is exquisitely sensitive to the local structure around the organic cation and the underlying vibrational motions. Contrary to crystallographic techniques providing information on time- and spatially averaged structure, spectroscopy enables access to atomic and molecular correlation functions in both space and time and, thus, furnish additional insights into the local structure and ensuing dynamics [75]. Moreover, these capabilities also find a natural ally in high-resolution solid-state NMR (ss-NMR) spectroscopy [146,147,148]. As illustrated by Drużbicki et al. [149], INS spectroscopy offers high potential to supplement ss-NMR in solving structural problems of a soft nature, particularly at cryogenic conditions which are hardly accessible to high-resolution NMR experiments (see, e.g., Ref. [150]). These synergies pave the way for the study of a wide variety of systems, including those disordered or nano-confined [151]. Drużbicki et al. [144] also highlight the mismatch between the averaged structures of OHPs defined with diffraction techniques and the local structure unveiled with spectroscopy. In particular, this work shows unequivocally that the commonly accepted *Pnma* model of the low-temperature phase of MAPbI${}_{3}$ is insufficient to describe the experimental spectroscopic data, pointing at the importance of octahedral deformations [144]. As extensively discussed in Ref. [75], the space-group assignment of each phase in this material has been debatable from the very beginning, and the origin of this discrepancy is linked to the dynamics and orientational disorder present at length scales commensurate with unit-cell dimensions. In this case, INS spectroscopy is particularly powerful.

The above work also illustrates the increasingly important role of first-principles modeling in linking the INS data to local structure. In this context, a very significant amount of progress has been made over the last decade with the development of advanced tools for the ab initio modeling of materials. These enable accurate and efficient Harmonic Lattice-Dynamics (HLD) calculations and makes more demanding, time-evolved modeling from first-principles (ab initio Molecular Dynamics, AIMD) feasible. A discussion on the current trends and practical aspects of modeling the low-frequency vibrational spectra can be found in Ref. [152]. Usually, electronic-structure calculations are performed using several implementations of density functional theory (DFT), relying either on the use of plane waves (e.g., CASTEP [153,154], QUANTUM ESPRESSO [155], ABINIT [156]), projector augmented-waves (e.g., VASP [157], GPAW [158]), and localized numerical (FHI-aims [159,160], SIESTA [161]) or Gaussian (CRYSTAL [162], TURBOMOLE [163]) basis sets, a hybrid plane-wave / Gaussian approach (CP2K [164]), or imposing all-electron full-potential Linearised Augmented Plane Waves (LAPW; WIEN2K [165] and ELK [166] programs). These codes offer calculations of phonon properties by means of finite-displacement methods (FD), typically using the Parlinski-Kawazoe Direct Method (DM) [167], or via perturbative treatments of ionic displacements through Density Functional Perturbation Theory (DFPT) [168,169,170]. Furthermore, the development of PHONOPY [171,172], an open source package for phonon calculations at the harmonic and quasi-harmonic levels with input from external codes (e.g., all the first-principles calculation codes listed above), should be emphasized. While the DM is straightforward to determine the total energy as a function of atomic displacements, phonon frequencies can be accurately calculated only at the wave vectors that are commensurate with the supercell geometry [173]. On the contrary, DFPT can calculate analytically the dynamical matrix at any arbitrary wave vector, but extensive programming might be required depending on the exchange-correlation (XC) functional and the basis-set definition used in the specific DFT implementation at hand [173]. The most extensive implementations of DFPT for lattice dynamics using a reciprocal-space formalism are offered by ABINIT [156], QUANTUM ESPRESSO [155] and CASTEP [154]. Recently, Scheffler and co-workers have released an efficient implementation using a real-space formalism, available in the FHI-aims code [160]. As an alternative, Lloyd-Williams and Monserrat [174] have proposed an extension of the FD method. This Non-diagonal Supercell Method (NCM) takes advantage of the periodicity of the system allowing a calculation of the dynamical matrix using supercells, which are much smaller than those used in DM calculations and, hence, more efficient. To date, a number of codes have also been developed to simulate neutron observables directly from HLD outputs [162,171,175,176,177,178,179,180].

Following the first high-resolution INS study of MAPbI${}_{3}$ by Drużbicki et al. [144], subsequent works have used TOSCA and VISION to interrogate the cation dynamics in OHPs. Using TOSCA, Kieslich et al. [181] investigated the relevance of the subtle balance between vibrational entropy and hybrogen bonding in the phase behavior of MAPbBr${}_{3}$. Mozur et al. have employed VISION to study the effects of Cs^+^ substitution in MAPbBr${}_{3}$ and FAPbBr${}_{3}$ perovskites [81]. These efforts are driven by the need to find new ways to improve the long-term stability and reliability of OHPs, cation mixing being an important route to this end. More recently, Drużbicki et al. [75] have used INS heavily supported by computational materials modeling to interrogate the mechanism behind phase stabilization in solid ${\mathrm{MA}}_{1-x}{\mathrm{FA}}_{x}{\mathrm{PbI}}_{3}$ mixed-cation compositions. This work provides a detailed and quantitative view of the local environment and vibrational dynamics of FA^+^ cations in this important OHP. In addition to confirming the formation of an orientational glass in FAPbI${}_{3}$, these results link the dynamical behavior of the cations in [MA/FA]PbI${}_{3}$ to the structural stabilization of the photophysically active perovskite phase via a novel locking mechanism. Detailed simulations validated by the INS data show the emergence of synergistic dynamics involving MA^+^ and FA^+^ cations, directly affecting the geometry of the surrounding inorganic framework. This work also illustrates the advantages of the joint use of INS and AIMD simulations to unravel the behavior of a massively disordered soft-matter system of technological relevance.

**Figure 6:**
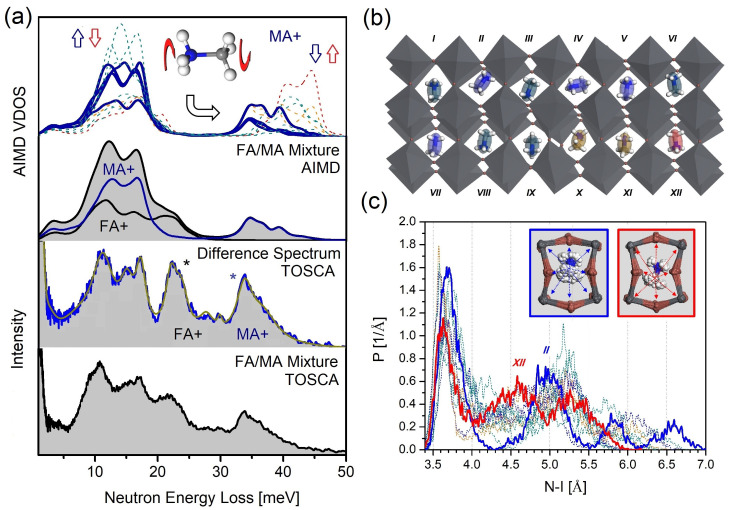
(**a**) The lower two panels show experimental INS spectra of the MA_0.4_FA_0.6_PbI${}_{3}$ solid-solution recorded on TOSCA+ at T = 10 K, along with the difference spectrum obtained by substracting the signal from FAPbI${}_{3}$, highlighting contributions from the MA^+^ cations [75]. These data are compared with the results of AIMD simulations in the upper two panels of (**a**). The theoretical INS spectrum of the MA_0.4_FA_0.6_ mixed-cation composition was constructed by stoichiometric weighting of the partial hydrogen-projected VDOS. MA^+^ cations were selected based on the analysis of the individual VDOSs for each of the twelve distinct types used in the structural model (see the bold curves in the top panel). The molecular model in (**a**) illustrates the lowest-energy internal mode of MA^+^. (**b**) Structural model used to describe the mixture, with FA^+^ omitted to ease visualization. Distinct MA^+^ cations are labeled with Roman numerals. (**c**) Distribution of closest nitrogen-iodine distances for each of the twelve MA^+^ cations. Two extreme cases are highlighted as bold curves (labeled as *II* and *XII*). Their time-evolved structures over a 30-ps interval are presented as cumulative models, as shown in the insets in (**c**). The same color coding has been used in all panels. [Unpublished data].

Figure 6 further illustrates the power of such a joint experimental-computational approach by presenting more recent time-evolved simulations, relying on an extensive model of [MA/FA]PbI${}_{3}$ solid solutions. MA^+^ cations are trapped inside the tetragonal perovskite framework of the parent FAPbI${}_{3}$ and are modeled and compared to the INS spectrum of the [MA/FA]PbI${}_{3}$ solid solution measured on TOSCA+. The analysis of the difference spectrum obtained by substracting the INS signal of the parent FAPbI${}_{3}$ from the [MA/FA]PbI${}_{3}$ mixture evinces strong changes in the vibrational dynamics of both MA^+^ and FA^+^ cations (the latter analyzed in detail in Ref. [75]). An excellent match between the experimental and simulated spectra gives us sufficient confidence to explore further the local structure around the MA^+^ cations. Such structure is characterized by a weakening of the hydrogen-bonding (H-bonding) motifs confining methylammonium cations in the surrounding iodoplumbate cages. As a result, MA^+^ undergoes a pronounced rattling behavior, locking the formamidinium cations in the neighboring voids, and preventing the transformation of the metastable perovskite framework to the thermodynamically stable hexagonal phase. Establishing the mechanism of stabilization of the perovskite phase (which from the outset stems from the softness of the iodoplumbate framework) can therefore facilitate the design of efficient routes for the further optimization of these solid solutions. In closing this discussion, we also note that to date a number of tools has been developed to support neutron scattering experiments with AIMD simulations, including calculations of the Vibrational Densities of States or VDOS (TRAVIS [182], MDANSE [183], OCLIMAX [184], DYNASOR [185]), as well as anharmonic phonon-dispersion relations at finite temperatures (DYNASOR [185], DynaPhoPy [186], phq [187]). Further discussion on the application of AIMD as a route to explore the vibrational dynamics of condensed matter is presented in Section 4.

## 3. From Confined Polymers to Soft Supramolecular Frameworks

**Figure 7:**
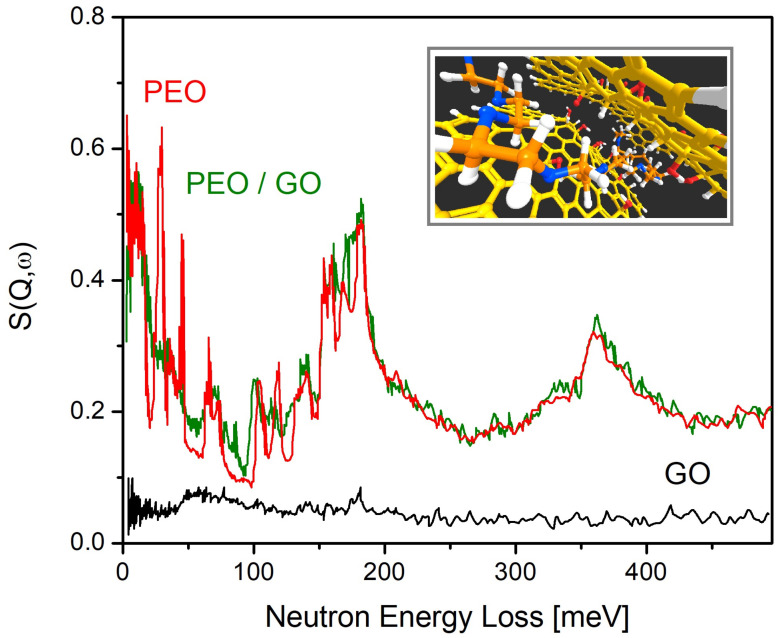
INS spectra of bulk (red) and confined (green) PEO. The black trace corresponds to the INS response of the graphite oxide substrate. Adapted from Ref. [202] with permission from The Royal Society of Chemistry.

The use of INS in the study of polymers has experienced a steady rise over the past decade, certainly facilitated by recent developments in the requisite instrumentation. Particular emphasis has been placed on exploring the consequences of spatial confinement at the nanoscale. The use of graphitic materials as confining media represents a nice illustration of the merits and strengths of INS to isolate the spectral response of the (hydrogenous) polymeric phase relative to that of the carbon-based substrate. Owing to the increased sensitivity of the new TOSCA+ spectrometer, for example, it has been recently shown that INS can be exquisitely sensitive to chemical composition and the underlying (and disordered) structure of graphene-related materials [188]. Considerable attention has also been given to the study of other carbon-based substrates with INS [189,190,191,192,193], including hydrogen spillover mechanisms [194,195] or the uptake and intercalation of atomic and molecular species, particularly water [196,197,198,199,200]. In this context, Romanelli et al. has used NCS to show that the confinement of water molecules within Graphite Oxide (GO) membranes has a ‘*soft*’ character, with the predominance of non-specific and weak interactions between water and the underlying nanostructured substrate [199]. In contrast, studies of organic polymers confined within carbon-based substrates reveals an intrinsically different picture [201]. Poly(Ethylene Oxide) (PEO) under extreme confinement was first studied using GO as a substrate [202]. The insights brought forward by the use of INS are evident from the data shown in Figure 7. This study served to establish the preferred conformation of the confined PEO phase, as well as to relate these structural changes to the strong suppression of collective phenomena, including crystallization and dynamical relaxation processes. Subsequent studies have exploited similar methodologies to investigate in detail a broad range of aspects linked to the above: molecular-size effects [203], intercalation versus adsorption [204], or the morphology and composition of the underlying substrate [205].

The study of hydrogen dynamics and associated confinement phenomena in Proton Exchange Membranes (PEMs) represents another timely example of the use of INS in the field of sustainable materials and associated technologies [206,207,208]. In a similar vein, there is also incipient activity in the development of hydrogen and other gas-storage materials. Carbon-based materials including fullerenes, nanohorns, or intercalation compounds constitute good examplars of soft media of increasing relevance for applications as gas stores, particularly hydrogen. In this regard, there are emerging opportunities for INS to provide unique insights into the mechanisms behind their hydrogen uptake at the atomic level [209,210], as well as for NCS as a unique analytical means to assess hydrogen levels quantitatively, as recently illustrated by Krzystyniak et al. [211].

Among the carbon-based materials that have been considered for hydrogen-storage, fullerene adducts continue to offer untapped opportunities. The full hydrogenation of the celebrated Buckminsterfullene molecule C${}_{60}$, for example, would lead to record-breaking hydrogen-storage capacities around 8 wt.% [212]. Several strategies have been attempted to ensure the reversibility of this process, either by intercalation [213,214,215,216,217,218,219,220,221,222,223,224,225,226,227,228,229,230,231,232], or through the use of intermediate hydrogen carriers, like ammonia [233,234,235]. The already excellent sorption properties of alkali-doped fullerides can be further improved via decoration with transition-metal nanoparticles [227,236]. An interesting feature of fullerides is also the possibility of their polymerization at extreme conditions [237,238,239,240], which opens new synthetic strategies for obtaining carbon allotropes with entirely new physico-chemical properties. Polymerization can also be promoted by the intercalation of a wide range of electron donors (e.g., metals including Li, Na, K, Rb, or Mg), by entering the voids of the host fullerene lattice to form fully fledged solids [237,241,242,243,244,245,246].

**Figure 8:**
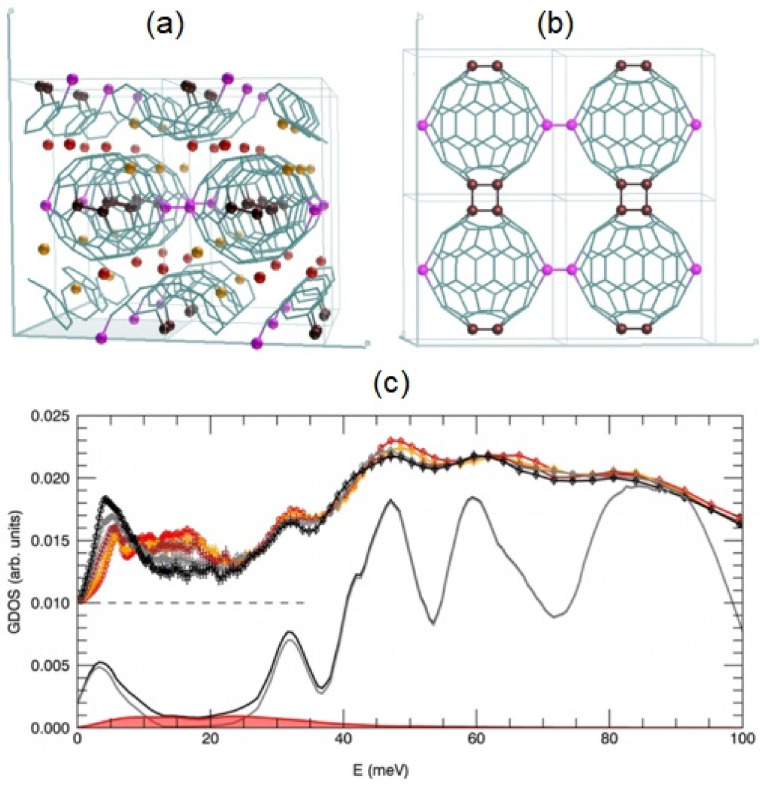
(**a**) Structure of Li_4_C_60_ obtained from DFT (VASP) geometry optimization within space group *I2/m*. Gray sticks are carbon bonds; magenta and brown spheres are carbon atoms involved in covalent intermolecular bonds, including [2 + 2] bridges and single bonds. Red and yellow spheres represent two different types of intercalated ions, Li_*T*_ and Li_*O*_, respectively. (**b**) View along the *c*-axis of one polymeric plane (the Li ions have been omitted for clarity). (**c**) (**Top**) Generalized Density of States (GDOS) derived from INS data collected between 300 K (polymer phase) and 700 K (monomer phase): red, 300 K; yellow, 610 K; brown, 630 K; gray, 640 K; and black, 700 K). (**Bottom**) GDOS extracted from the MD simulations at 800 K (see text) in the monomer phase (black solid lines, total GDOS; gray solid line, carbon GDOS; red area, lithium GDOS). The INS spectra were collected on Mibemol (LLB, Saclay, FR). Reproduced from Ref. [246] with permission from APS (https://doi.org/10.1103/PhysRevLett.113.215502) (accessed on 28 April 2021).

As an illustration of this line of work, the structure and dynamics of the fullerene polymer Li_4_C_60_ was studied with INS spectroscopy by Rols et al. [246,247]. Representative results are illustrated in Figure 8. The use of ND and INS, combined with extensive ab initio modeling allowed the authors to solve its solid-state structure. Figure mattiaa–b show the rather exotic structure of this polymeric material, leading to the rich temperature dependence of INS features displayed in Figure 8c. Further, it has found that the intercalated ions exhibit partial disorder, as further corroborated by the analysis of the INS data. The strength and the nature of the peculiar polymeric bonding between the sub-units rely on a three-electron charge transfer from the Li atoms to every C_60_ molecule. In addition to its good hydrogen-storage capabilities, the system shows superionic conductivity at low temperature, which is an exceptional property for a solid material, therefore hinting at new applications [248]. The calculations presented in this work also predict an unstable Li sub-lattice, a finding that is consistent with the aforementioned ionic conductivity. The temperature dependence of INS features depicted in Figure 8 c is also sensitive to the depolymerization transition, characterized by a (rather counterintuitive) transfer of spectral weight to lower frequencies in the monomeric phase [246].

Nanoporous coordination compounds and their polymeric counterparts constitute yet another family of soft media with new properties and great potential for applications. To date, Metal-Organic Frameworks (MOFs) and Covalent-Organic Frameworks (COFs) have been studied in greater depth. Owing to its superb sensitivity to hydrogen, INS has become a powerful probe of the structural and gas-sorption properties of these materials, and its use has certainly proliferated in recent years. Table 1 provides a comprehensive summary of work to date. Following the seminal papers from Eckert and co-workers [249,250], INS has evolved as a leading technique providing insights on the binding and dynamics of molecular hydrogen (primarily in its *para* form, *p*–H_2_) absorbed by MOFs. As illustrated by a growing number of works [209,210,251,252,253,254,255,256,257], the use of intense beams of low-energy neutrons is ideally suited for the study of ro-vibrational excitations in H_2_. In particular, the last decade has witnessed unprecedented progress in MOF research using INS, along with a considerable broadening in scope beyond H_2_ uptake, which is still ongoing (see, e.g., Ref. [258]). Alongside, instrument developments at neutron sources have continued to support these research programmes, including: (i) a new Target-Station 2 (TS2) at ISIS, which has considerably improved the flux on TOSCA at low-energies; (ii) developments in sample environment equipment, including advanced gas-handling capabilities (see, e.g., Ref. [259]); (iii) ongoing developments in indirect-geometry instruments, reducing the requisite sample quantities considerably [137,138,139,140,141]; (iv) the commissioning of new instruments, like VISION at the SNS and LAGRANGE at the ILL [143], or the design of VESPA for the ESS [1]; and (v) progress in the field of ab initio modeling and its integration with experiments—as shown in Table 1, we underline the substantial increase over time in the number of publications using solid-state DFT. Altogether, these have enabled the study of the vibrational dynamics over a wide energy-transfer range and, more importantly, to place a fresh focus on the structural properties and flexibility of these soft materials, key to understand their rich polymorphism and sorption behavior [74,260]. As shown by Ortiz et al. [261], some flexible MOFs (e.g., MIL-53 or DMOF-1) show highly anisotropic elastic properties, with deformation directions exhibiting very low Young’s and shear moduli, approaching a 400:1 ratio between the most rigid and the softest direction. Negative compressibilities as a function of temperature or pressure also seem to be the norm rather than the exception in these materials.

**Figure 9:**
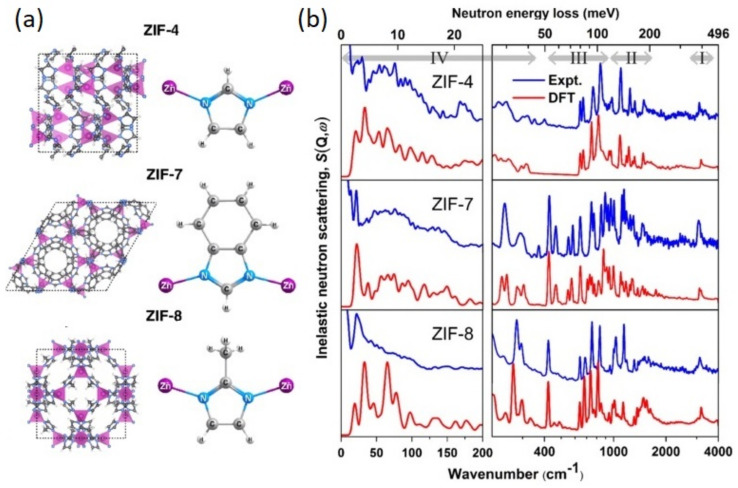
(**a**) Nanoporous hybrid framework structures of ZIF-4, ZIF-7, and ZIF-8. The inorganic building blocks are represented by purple ZnN_4_ tetrahedra. (**b**) Experimental (TOSCA T = 10 K) and theoretical (DFT; CRYSTAL14) INS spectra for these compounds. Adapted from Ref. [264] with permission from APS (https://doi.org/10.1103/PhysRevLett.113.215502) (accessed on 28 April 2021).

The outcome of the above-discussed developments can be illustrated with the family of Zeolitic Imidazolate Frameworks (ZIFs). ZIFs are nanoporous materials comprised of tetrahedrally coordinated nodes and imidazole-derived organic linkers. The linkage between the metal and imidazole resembles the Si–O–Si angles present in zeolites, which explains their name. Their facile synthesis, high stability under ambient conditions, and commercial availability of the building blocks make them one of the most studied classes of MOFs regarding their mechanical properties [260]. For instance, ZIF-8 has a remarkably low shear modulus below 1 GPa [262], claimed to be the lowest value known for a crystalline solid. It is also interesting to note that some of the OHPs discussed earlier [113] have very similar values of *C_44_* as those seen in ZIF-8. Further, ZIF-4 is also known to exhibit a remarkably rich polymorphic behavior at ambient and high pressures, and this phenomenology is certainly linked to the flexibility of its network structure at the nanoscale [260,263]. Figure 9 presents the INS spectra of a ZIF series [264], obtained on a previous incarnation of TOSCA, along with the results of accurate (optimized for the THz range) HLD calculations performed with the CRYSTAL code. This figure illustrates the synergy between the high-resolution INS experiments and theoretical predictions, allowing the full and quantitative interpretation of a rather complex vibrational spectrum over the entire range [264]. The low-energy part of the INS data is of particular relevance to understand the unexpected sorption properties of these materials. The large cavities interconnected by narrow and flexible channels characteristic of these ZIFs lead to novel molecular-sieving phenomena driven by low-energy modes beyond those at play with small molecules like H_2_ or CH_4_. These dynamical processes can explain their rather unexpected ability to absorb larger molecules which would not ‘*fit-in*’ by considering the rigid structure of the substrate [265]. At an atomic level, this so-called ‘*gate-opening*’ is related to the swing of the imidazolate linkers, thereby increasing the intrinsic porosity. Developments in gas-handling systems have also paved the way to probe gate-opening and related network-breathing phenomena arising from the unique elastic properties of MOFs—see entries in Table 1 and Refs. [151,266,267,268,269,270,271,272,273,274,275,276,277,278,279,280,281,282,283,284,285,286,287,288,289,290].

**Table 1:** Summary of INS studies on MOFs and COFs. The first column indicates the acronym of the material, as commonly used in the literature. The second column indicates whether the focus of the investigation was on the bare material or on the uptake of the chemical species shown. The third and fourth columns show the energy-transfer range and INS spectrometer used (QENS, IPNS; FANS, NIST; FOCUS, SINQ; TOSCA and MARI, ISIS; TOFTOF, MLZ; IN5, ILL; VISION and SEQUOIA, SNS). Asterisks on the latter column indicate that solid-state DFT was used to interpret the INS data. Works are presented in chronological order, with the year of publication given along with its bibliographic reference in the last column.

Acronym	Investigation	Range	Instrument	Reference
MOF-5	*p*–H_2_	0–20 meV	QENS	2003 [249]
IRMOF-X (X = 1, 8, 11, 177)	*p*–H_2_	0–20 meV	QENS	2005 [250]
MOF-5	Bare material	20–170 meV	FANS *	2006 [291]
NaNi_3_(OH)(SIP)2	*p*–H_2_	0–20 meV	QENS	2006 [292]
HKUST-1	*p*–H_2_	5–45 meV	FANS	2007 [293]
TMBB	*p*–H_2_	0–30 meV	QENS	2007 [294]
MIL-53	Bare material	50–180 meV	FANS *	2008 [295]
MOF-74	*p*–H_2_	5–25 meV	FANS	2008 [296]
PCN-12	*p*–H_2_	0–20 meV	QENS	2008 [297]
PCN-6; PCN-6’	*p*–H_2_	0–20 meV	QENS	2008 [298]
HKUST-1	*p*–H_2_	5–200 meV	FANS *	2009 [299]
ZMOFs	*p*–H_2_	0–25 meV	QENS	2009 [300]
CPO-27-M (M = Ni, Co, Mg)	*p*–H_2_	0–15 meV	FOCUS	2010 [301]
Cr_3_(BTC)_2_	*p*–H_2_	5–45 meV	FANS	2011 [302]
Mg_2_(dobdc)	*p*–H_2_	5–45 meV	FANS	2011 [303]
MOF-324	*p*–H_2_	0–25 meV	QENS	2012 [304]
rht-MOF-1 and rht-MOF-4a	*p*–H_2_	0–20 meV	FOCUS	2012 [305]
Fe_2_(dobdc) and Fe_2_(O_2_)(dobdc)	*p*–H_2_	0–125 meV	FANS / TOSCA	2012 [306]
NU-301 and NU-302	*p*–H_2_	0–20 meV	FOCUS	2013 [307]
HKUST-1	*p*–H_2_	0–50 meV	TOSCA / MARI	2013 [308]
MIL-53(Fe)	CH_3_OH	0–250 meV	TOSCA *	2013 [309]
rht-MOF-7	*p*–H_2_	0–20 meV	TOFTOF	2013 [310]
CPO-27–M (M = Mn, Cu)	*p*–H_2_	0–20 meV	TOFTOF / FOCUS	2014 [311]
SIFSIX-2-Cu and SIFSIX-2-Cu-i	*p*–H_2_	0–20 meV	TOFTOF / FOCUS	2014 [312]
Y-FTZB	*p*–H_2_	0–20 meV	TOFTOF	2014 [313]
NOTT-300	*p*–H_2_	0–250 meV	TOSCA *	2014 [314]
ZIF-4, ZIF-7, and ZIF-8	Bare material	0–400 meV	TOSCA *	2014 [264]
a-[Mg_3_(O_2_CH)_6_]	*p*–H_2_	0–20 meV	QENS	2015 [315]
Zn(trz)(tftph)	*p*–H_2_	0–20 meV	TOFTOF	2015 [316]
M-MOF-74 (M = Mg, Ni, Co, Zn)	*p*–H_2_	0–20 meV	FOCUS	2015 [317]
NOTT-300	C_2_H_6_, C_2_H_4_, and C_2_H_2_	0–250 meV	TOSCA *	2015 [314]
M-soc-MOF-1-X (M = Fe, In; X = a, b)	*p*–H_2_	0–20 meV	IN5	2016 [318]
ZIF-8; ZIF-8@AC	N_2_	0–150 meV	VISION *	2016 [266]
Cu(I)-MFU-4l	H_2_ and D_2_	0–40 meV	VISION *	2016 [267]
MFM-300(In)	H_2_ and CH_4_	0–250 meV	TOSCA *	2016 [268]
MFM-300(In)	N_2_, CO_2_, and SO_2_	0–250 meV	TOSCA *	2016 [269]
ZIF-8	D_2_O and CH_4_	0–200 meV	TOSCA	2016 [266]
COF-1; COF-2	*p*–H_2_	0–20 meV	QENS	2017 [270]
ZIF-7	N_2_	0–200 meV	VISION *	2017 [271]
ZIF-8	N_2_, O_2_, Ar, and CO	0–150 meV	VISION	2017 [272]
MFM-300(Sc)	I_2_	0–250 meV	TOSCA *	2017 [273]
MFM-300(V)	CO_2_	0–250 meV	TOSCA *	2017 [274]
MIL-140A	Bare material	0–250 meV	TOSCA *	2017 [275]
MFM-305 and MFM-305-CH_3_	CO_2_	0–250 meV	VISION *	2018 [276]
MFM-102-NO_2_	C_2_H_2_	0–250 meV	VISION *	2018 [277]
MFM-520	NO_2_	0–250 meV	VISION *	2019 [276]
Zn(MeIm)_2_	Bare material	0–500 meV	SEQUOIA	2019 [319]
MFM-102-NO_2_ and MFM-102-NH_2_	CO_2_	0–250 meV	VISION *	2019 [278]
ZIF-4	Bare material	0–250 meV	VISION *	2019 [279]
${\left[Cu24\left(OH-mBDC\right)24\right]}_{n}$	CO_2_	0–250 meV	TOSCA	2019 [280]
ZIF-4(Zn)	Bare material	0–250 meV	TOSCA *	2019 [281]
ZIF-7	CO_2_	0–150 meV	TOSCA	2019 [282]
HKUST-1	Drug encapsulation	0–250 meV	TOSCA *	2019 [151]
MFM-126	CO_2_	0–400 meV	TOSCA*	2019 [283]
MFM-100	Benzyl alcohol	0–250 meV	TOSCA*	2019 [284]
Pd@OX-1	Catalytic properties	0–400 meV	TOSCA*	2019 [285]
MFM-170	H_2_O and SO_2_	0–150 meV	TOSCA *	2019 [286]
Y-shp-MOF-5 and Cr-soc-MOF-1	D_2_O and CH_4_	0–300 meV	VISION *	2020 [287]
MIL-100 (Fe)	Drug Encapsulation	0–250 meV	TOSCA	2020 [288]
MFM-520	D_2_O, CO_2_, and SO_2_	0–250 meV	TOSCA *	2020 [289]
MFM-300(M) (M = Al, Fe, V^III^, V^IV^)	NH_3_	0–200 meV	TOSCA / VISION *	2021 [290]

**Figure 10:**
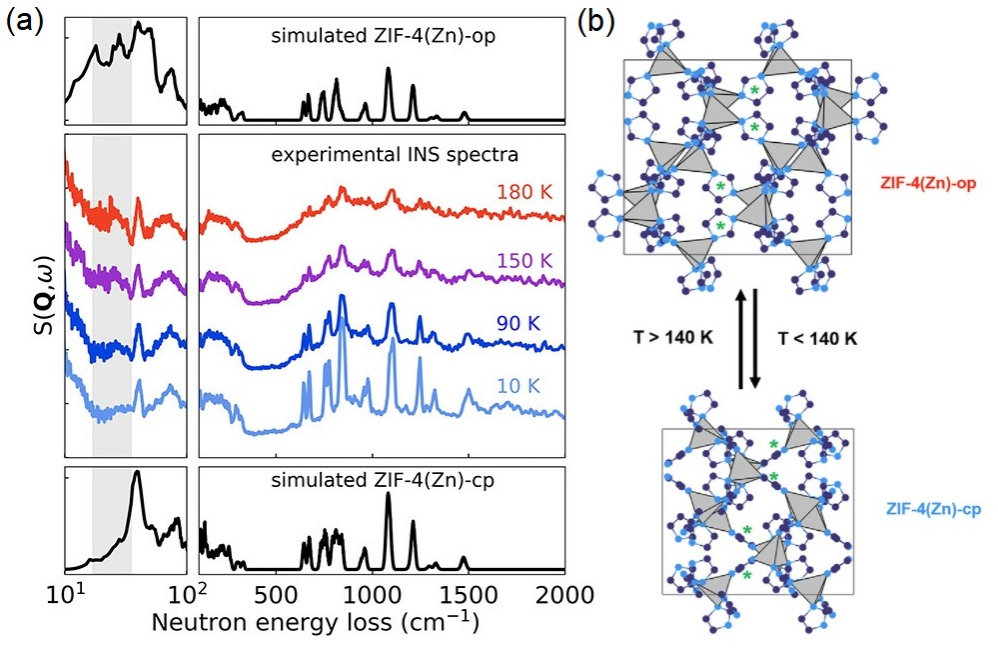
(**a**) Experimental (TOSCA+) and simulated (DFT; VASP) INS spectra of closed- (cp) and open-pore (op) ZIF-4(Zn) structures. (**b**) Schematic diagram of the low-temperature structure of ZIF-4(Zn)-cp and the high-temperature structure ZIF-4(Zn)-op, looking down the *b*-axis in both phases. A volume contraction of approximately 24% associated with the cp-to-op phase transition is related to a rotation of the imidazolate linkers. Four of such rotations are highlighted by green stars. Reprinted with permission from Ref. [281]. Copyright (2021) American Chemical Society.

The aforementioned developments in instrumentation, sample environment, and ab initio modeling can be further illustrated by considering the polymorphism exhibited by ZIF-4 at ambient pressure. Figure 10 presents the INS spectra as a function of temperature, measured on the TOSCA+ spectrometer [138,139,140]. Owing to a greatly improved sensitivity, it is now possible to explore phase diagrams across physical space, alongside theoretical modeling. While extremely rare a decade ago [291], phonon calculations are now becoming an inseparable part of structural studies on MOFs using INS (see Table 1), allowing to decode complex vibrational information delivered by these metal-organic frameworks. However, as noted by Vanpoucke et al., the computations with periodic DFT are still challenged by the flexibility of these materials, and the guidelines from experience with standard solid-state calculations cannot be simply transferred to flexible porous frameworks [320]. As shown by Butler et al. [281] (see Figure 10), variable-temperature high-resolution INS experiments give us a direct handle on changes to the lattice dynamics across the closed-to-open-pore phase transition in ZIF-4. Moreover, these data allow for a robust validation of the calculations performed within the Quasi-Harmonic Approximation (QHA), one of the methods of choice in the study of thermal expansion from first-principles. Using this methodology, the authors were able to identify the key structural and dynamical features that govern the free-energy landscape of the material, including the central role played by the vibrational entropy as a driving parameter for their exceptional flexibility.

## 4. Back to Basics: Water

Water continues to be the focus of attention of many, and some recent works have capitalized from developments in neutron spectroscopy to move beyond the state-of-the-art. Following the discovery of ice XV (α-XV) by Salzmann et al. [321], the recent identification of ice XIX [322] confirms that the condensed phases of water are far from being a solved challenge [323], and have also stimulated developments in both neutron scattering [324] and computational modeling from time immemorial [325]. In this regard, neutron scattering constitutes a priceless tool for the ultimate validation of the theoretical approaches developed to describe both structure and dynamics, from this seemingly simple triatomic system to the complex materials described earlier in this review. One of the most celebrated examples is perhaps the total-scattering experiments by Soper et al., providing the Radial Distribution Functions (RDFs) of water and ice [326,327] to validate a countless number of theoretical approximations. In addition to diffraction data, calculations of the VDOSs based on empirical or ab initio approaches can be considered as the most effective and direct way of testing our description of interatomic and intermolecular interactions, indispensable to build increasingly accurate models.

**Figure 11:**
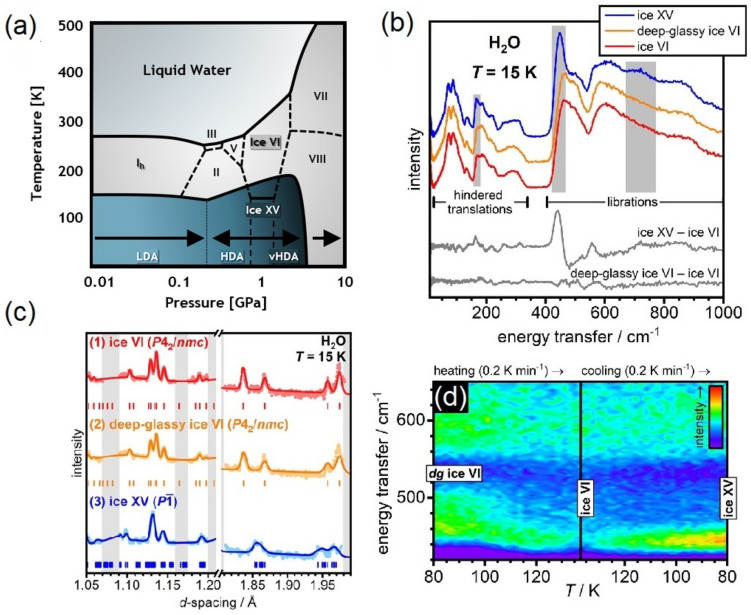
(**a**) Phase diagram of H${}_{2}$O in the range T = 4–300 K and *p* = 0.01–10 GPa, highlighting the thermodynamically stable forms of ice of relevance to our discussion (crystal phases VI and XV). Ice XV is the hydrogen-ordered counterpart of ice VI and is thermodynamically stable below 130 K over the pressure interval 0.8–1.5 GPa [321]. The phase diagram also includes the ranges of observation of metastable amorphous ices (Low-, High-, and very-High-Density Amorphous forms are denoted as LDA, HDA, and vHDA, respectively). The LDA phase is mostly found in the low-pressure region of the phase diagram (0–0.2 GPa), whereas HDA and VHDA occur at intermediate pressures (0.2–2 GPa). At high pressures (>2 GPa), only crystalline phases are stable [350]. (**b**) INS spectra of ice VI, deep-glassy ice VI, and ice XV, collected at T = 15 K on TOSCA+. The gray-shaded areas highlight the spectral range where major differences between ice VI and XV are observed. The spectra are shifted vertically for clarity. Difference spectra are shown in the lower part of the panel [328]. (**c**) ND patterns of (1) ice VI, (2) deep-glassy ice VI, and (3) ice XV collected simultaneously on TOSCA+ at T = 15 K. The experimental diffraction data are shown as light data points and the associated Rietveld fits as darker solid lines. Tick-marks indicate the expected positions of Bragg reflections. [328]. (**d**) Contour plot of the librational region upon heating deep-glassy ice VI from 80 to 138 K, followed by cooling back to 80 K [328]. Adapted with permission from Refs. [328,350]. Copyright (2021) American Chemical Society.

The study of water with INS has a long and distinguished history, providing insights into the nuclear dynamics of a large fraction of the known ice phases [328,329,330,331], water at extreme conditions [332], and under ultra-confinement [333,334]. Figure ice-polymorphisma shows the phase diagram of water, highlighting both crystalline and amorphous phases. An exhaustive discussion of this phase diagram (including metastable forms of ice not shown in this figure) can be found in Ref. [323]. Ice XV can be considered as a hydrogen-ordered polymorph of form VI, revealing an antiferroelectric ordering of protons. However, it has been recently shown that for the unit cell of ice VI, a total of forty-five different ferroelectric or antiferroelectric types of order are possible within the remit of the ice rules, clearly illustrating that there are more new phases expected to be discovered in the future [335]. The discovery of form XIX (β-XV) tends to confirm these notions, yet not without debate [336,337]. Rosu-Finsen and Salzmann have suggested that ice β-XV is not a crystalline form of ice, but that it rather contains an immobile disordered network of hydrogen atoms, called a deep-glassy state [336,337,338]. Using per-deuterated ice XV, Gasser et al. has argued in favor of the existence of β-XV [322]. However, INS experiments performed on hydrogenous samples of ice VI, ice XV, and deep-glassy ice VI (or ice β-XV above) by Rosu-Finsen et al. [328] question this conclusion. Figure ice-polymorphismb displays the INS spectra recorded for each phase, unequivocally illustrating striking similarities between the spectral envelopes of ice VI and deep-glassy ice VI. This debate also raises further questions on the importance of isotopic effects and their relation to the stability of the phase of interest. The most-recent INS experiments on ice polymorphs by Rosu-Finsen et al. [328] also illustrate the state-of-the-art developments on the TOSCA+ instrument (see Figure 11b–d), providing up to a two-order-of-magnitude increase in count rates, and enabling parametric neutron-spectroscopic studies alongside simultaneous ND measurements [137,138,139,140,141]. Figure ice-polymorphism also confirms that the ND profiles of both ice VI and deep-glassy ice VI are remarkably similar and both data sets could be fitted well using the same model of the long-range order as ice VI, strikingly different from the ND profile of the XV phase. Finally, an overwhelming increase of the sensitivity of TOSCA+ allows to complement the calorimetric studies by real-time INS measurements as a function of temperature (see Figure 11d.) upon heating deep-glassy ice VI from 80 to 138 K, followed by cooling to 80 K. These in-situ experiments provide further insights into the thermodynamic relation between ice polymorphs and it is anticipated that a similar approach will be deployed in the future to resolve remaining and emerging discrepancies across the phase diagram of water.

**Figure 12:**
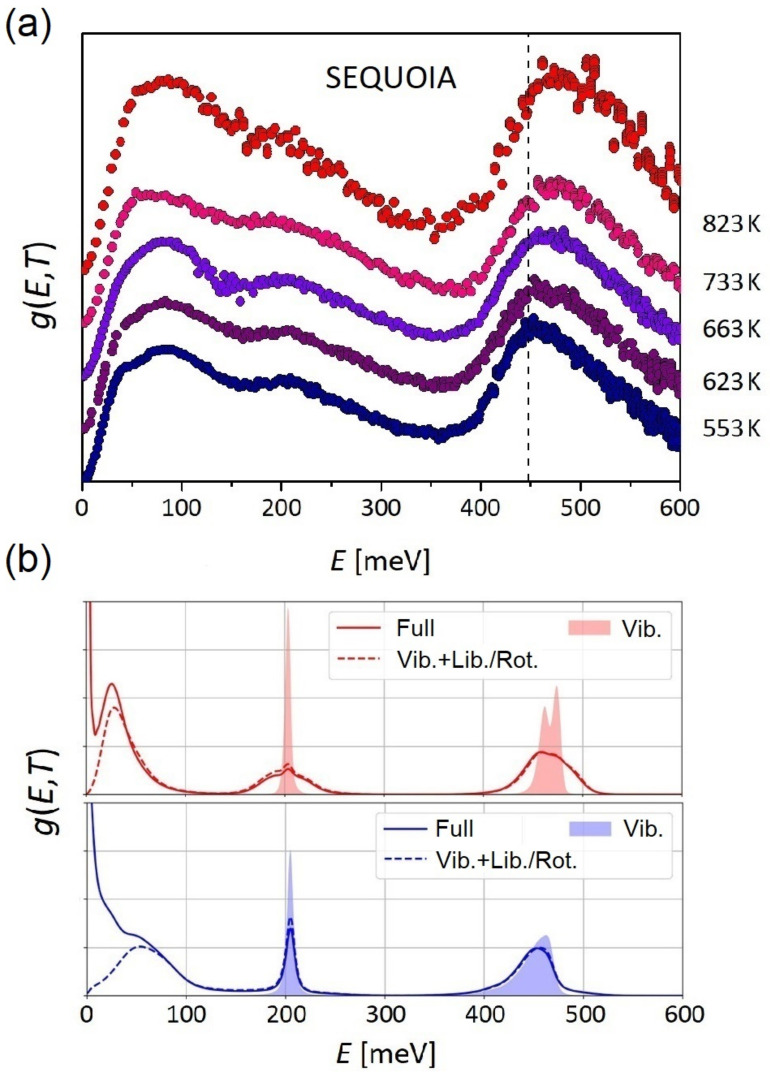
(**a**) Hydrogen-projected VDOSs as a function of temperature at a pressure of 0.025 GPa. These data have been normalized to unity and vertically offset to facilitate visual comparison. (**b**) Simulated VDOSs of H_2_O at subcritical (bottom) and supercritical (top) conditions. In each case, the full spectrum (solid line) is compared with the projected VDOS (dashed line), after removing translational contributions. The shaded spectra correspond to intramolecular vibrational spectra, with librational and rotational motions projected out. Adapted with permission from Ref. [332]. Copyright (2021) American Chemical Society.

The superb ability of INS to elucidate local structure is not limited solely to fully or partially ordered solids, as illustrated by recent state-of-the art studies of supercritical water (SCW). SCW is formed at 647 K and 0.0221 GPa, and its thermophysical properties are quite different from water at normal conditions [147,339]. There is an increasing interest in this exotic state, of promise for many sustainable and industrial applications, including gasification of biomass [340], construction of new-generation Supercritical-Water-Cooled Reactors (SCWRs) [341], or as a novel medium for soft matter research [342]. However, a detailed picture of the structure of water at these extreme conditions, as well as its link to unique physico-chemical properties remain unclear. Parrinello and co-authors have studied water at supercritical conditions from first-principles [343], showing that contrary to the ordinary liquid state, the H-bond network is destabilized to various extents. There is a continuous breaking and forming of anomalous H-bonded structures and no continuous network per se, significantly affecting the dielectric response. This fundamental study has been recently extended by Andreani et al. [332], providing a detailed structural picture of this fascinating system. This study capitalized from a combination of high-resolution experiments as a function of temperature and state-of-the-art AIMD, as presented in Figure 12. The work by Andreani et al. [332] has two important implications. First, it features INS and NCS as powerful probes of the temporal and spatial characteristics of H-bonding interactions. And second, it puts state-of-the-art AIMD to the test using the latest experimental and computational methodologies.

**Figure 13:**
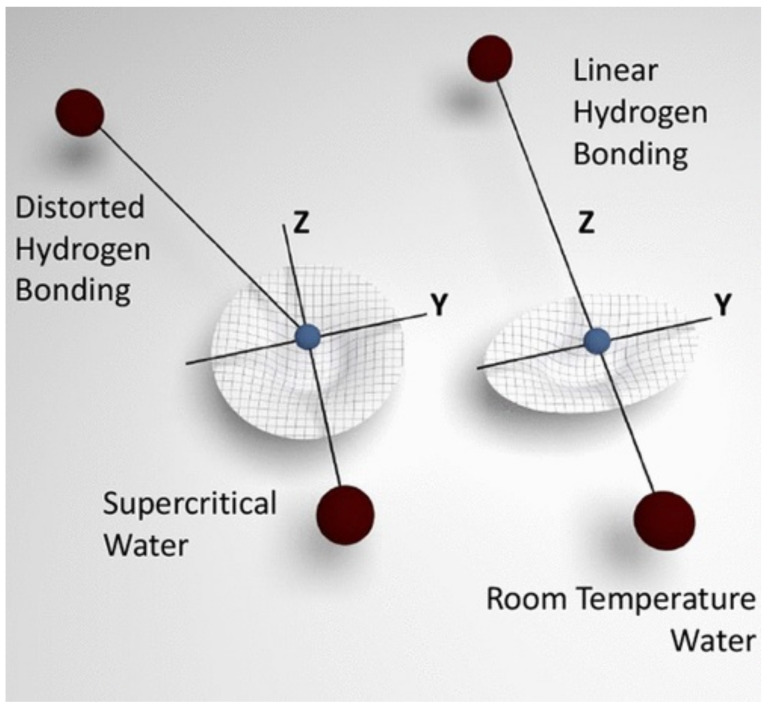
Pictorial representation of the local H-bond geometry for H_2_O at supercritical (**left**) and ambient conditions (**right**). The figure depicts the coordinate frames used in the interpretation of NCS data, and the corresponding (partially isotropic and anisotropic) proton momentum distributions. Reprinted with permission from Ref. [332]. Copyright (2021) American Chemical Society.

In addition to challenging INS experiments, NCS has been used to provide additional and much-needed information on the local potential around water molecules in the supercritical state [40,344,345,346,347,348,349]. Andreani et al. has identified short-lived water clusters in SCW [332], thus demonstrating that the technique is sensitive to local dynamics in the femtosecond time scale. On the basis of combined experiments and simulations, the authors have been able to show how H-bonds decrease in number as a function of temperature under constant pressure, and have revealed a pronounced distortion of H-bonds in these short-lived water clusters, which increases the coupling of intermolecular and intramolecular vibrations at supercritical conditions. This coupling modifies the H-bonding potential exerted on hydrogen, making it less anisotropic in the molecular plane compared with water at ambient conditions. This microscopic picture of the local environment of water molecules in SCW is displayed in Figure 13.

In addition to these experimental efforts, one also needs to note the accurate computational modeling using AIMD, building upon the seminal work of Car-Parinello [351]. Recent and exciting developments include those from Artificial Intelligence (AI) and the use of Deep Neural Networks (DNNs) to represent the interatomic potential [352]. Such an approach, as well as that by Behler and Parinello [353], allows for a description of highly dimensional potential-energy hypersurfaces in systems of arbitrary size whilst retaining the accuracy of high-end electronic-structure calculations. The end result is a speed-up of an otherwise very costly AIMD simulation by several orders of magnitude relative to the high-end reference method from which the artificial neural network is trained. These calculations of interatomic potentials and forces are fully sufficient to deal with the dynamic information encoded in neutron-scattering experiments. Hence, it also paves the way for the use of these methodologies to describe the longer timescales probed with QENS or NSE. INS, with its direct link to nuclear dynamics, becomes the method of choice for the validation of such theoretical developments. In addition, we note that recent progress in the development of Machine Learning (ML) ML-based simulations includes the calculation of charges, dipole moments and polarizability tensors, of direct relevance to the description of experimental observables accessible with optical spectroscopies [354,355]. Another area of increased interest relates to the use of ML algorithms in the calculation of molecular wavefunctions [356].

The spectacular speed-up of MD simulations with ML-trained Force-Fields (ML-FF) also helps us formulate and tackle important questions concerning which parent electronic-structure method is most appropriate to describe structural and dynamical properties. And, in this context, water serves as an obvious benchmark. Recent advances employ the gold-standard of quantum chemistry, i.e., Coupled-Cluster (CC) theory, yet still limited to small-molecules, like water and molecular clusters [357,358]. To date, DFT calculations on extended systems still remain as the work-horse. In the aforementioned work on SCW by Parinello and co-authors [343], the authors used the Generalized-Gradient Approximation (GGA) to the XC functional, which, to date, remains the most-commonly used approach in simulations of condensed-matter systems. Although considerable progress has been made with the construction of hybrid XC functionals or parallel developments in advanced semi-empirical corrections accounting for van-der-Waals (vdW) contributions, progress in constructing a better functional obeying a number of required constrains and norms for semi-local DFT has been far more modest. The development of a Strongly-Constrained and Appropriately Normed (SCAN) functional of a meta-GGA form [359] certainly offers a way forward, significantly improving accuracy when calculating the properties of different types of bonding [359,360,361]. SCAN provides an accuracy comparable to hybrid functionals at a considerably reduced cost, while still remaining a pure DFT functional by definition [362]. Both SCAN and hybrid functionals may be, therefore, be regarded as the most-advanced and still computationally feasible approximations to describe extended condensed matter systems. In the case of the structure of liquid water, its underlying H-bond geometry, and associated Mean Square Displacements (MSDs), Chen et al. [363] have shown that SCAN outperforms GGA. LaCount and Gygi have further confirmed the superb performance of SCAN in the description of the VDOSs of liquid water at ambient conditions [364]. This finding has been corroborated by AIMD simulations of liquid water using high-quality meta-GGA functionals presented by Ruiz-Pestana et al. [365]. The work by Andreani et al. [332] relied on SCAN-trained DNN potentials, fully confirming their robustness and high reliability to study in a realistic manner the structure and dynamics of water at supercritical conditions where—to first order—nuclear quantum effects (NQEs) may be ignored in these high-temperature simulations. The role of NQEs cannot be, however, neglected when discussing water at ambient conditions and below. Due to the small mass of the proton, NQEs, such as spatial nuclear delocalization, zero-point energy (ZPE), and proton tunneling, modify the H-bond geometry and strength in a significant manner [366]. In recent years, we are witnessing a pronounced interest in these effects, which can the key for a better understanding of fundamental phenomena and macroscopic properties. An exhaustive discussion can be found, e.g., in Refs. [367,368,369,370,371].

**Figure 14:**
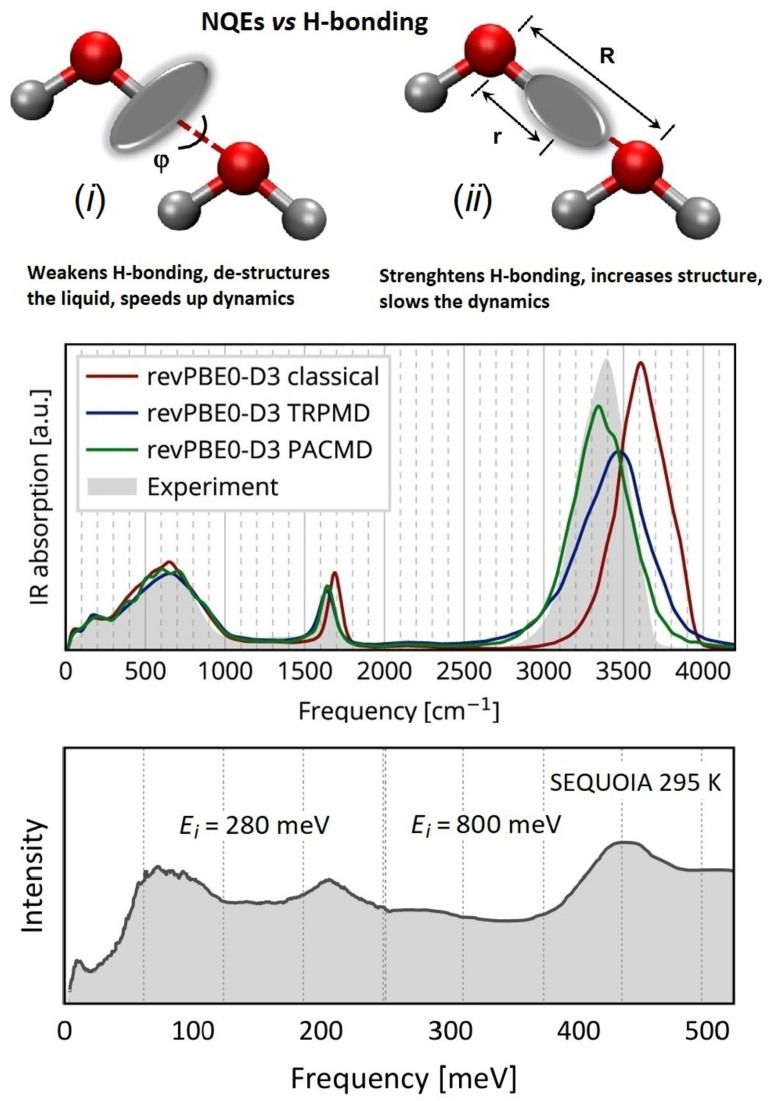
Bottom: experimental INS spectrum of liquid H_2_O measured on SEQUOIA at ambient conditions. Two different incident energies have been used, as indicated in the figure [388]. Middle: experimental IR spectrum of H_2_O at ambient conditions, along with the results of AIMD predictions using vdW-corrected hybrid-DFT (revPBE0-D3 ML-FF) and different computational schemes—classical MD and approximate path-integral simulations (TRPMD and PACMD) [376]. Top: cartoon illustrating competing quantum effects in the H-bonding between two water molecules. There are two qualitatively different contributions to the vibrational ZPE: (*i*) the one arising from the two bending vibrational modes, in the plane of the water molecule and perpendicular to it (not shown); (*ii*) a second one associated with the O–H stretch. As the O–O distance R decreases, the contribution of the stretch decreases, and that of the bend increases. Consequently, the two contributions weaken and strengthen the intermolecular H-bond, respectively. [367]. Adapted with permission from Refs. [367,376,388]. Copyright (2021) American Chemical Society.

To illustrate the above in the particular case of water, calculations of vibrational properties using the GGA approximation seem to provide the ‘*right*’ answer, yet it has been found that this agreement is caused by a fortuitous cancellation of fairly large intrinsic errors in the description of the potential energy surface by these functionals [372,373]. In addition, most simulations to date do not incorporate NQEs from the outset, including ZPE contributions to the free energy which may be particularly pronounced for light species. These effects have been illustrated by Ruiz-Pestana et al. [365], showing that well-justified approximations, like high-quality meta-GGA or hybrid functionals, give a worse description of vibrational properties than the simpler GGA scheme when NQEs are not taken into account [365]. More simple scaling corrections have been proposed for practical reasons [374], yet these should always be assessed carefully to undertake physically meaningful simulations, particularly in those systems where NQEs are expected to play a significant role. This cautionary note is confirmed by the works of Ruiz-Pestana et al. [375] and Marsalek et al. [376], showing that the inclusion of NQEs using advanced meta-GGA DFT functionals can reproduce the properties of bulk water including RDFs, MSDs, and vibrational spectra (IR and Raman) with an accuracy comparable to those of far-more-costly hybrid functionals. Figure 14 serves to confirm the above considerations, with an example of the infrared spectrum of water simulated with both classical and approximate quantum MD simulations using the vdW-corrected hybrid functional (revPBE0-D3) [376]. While classical simulations are unable to describe satisfactorily the bending (*ca.* 1600 cm^−1^/200 meV) and stretching regions (*ca.* 3500 cm^−1^/450 meV) of H-bonded water, the quantum simulations provide the results with an excellent match to the experimental spectrum at ambient conditions. The cartoons in this Figure illustrate the competing quantum effects at play in the H-bonding between two water molecules. These affect the bending and stretching modes, with two qualitatively different contributions to the vibrational ZPE [367]. The left-upper panel in the Figure depict the in-plane bending vibrational mode. The right cartoon shows the O–H stretch. These contributions weaken and strengthen the H-bond, respectively, further refining the structural picture drawn from the RDFs accessible from diffraction. In addition, the results from IR spectroscopy (middle panel) are confronted with the INS spectrum of ambient water measured with the SEQUOIA spectrometer (see bottom panel). A small difference in the peak position of the OH-stretch band probed with IR and INS can be noticed. In this regard, INS spectroscopy should be considered as a better suited approach for the validation of theoretical predictions, as it is directly related to the underlying VDOS. On the contrary, accurate simulations of both the position and the shape of a band observed in IR spectroscopy requires an accurate treatment of dielectric or other charge-derived properties, including long-range dipole couplings [377] and electrical anharmonicities [378]. As illustrated in the Figure, NQEs can be successfully accounted for using quantum simulations. In its simplest formulation, well-established classical methods can be used to properly map the properties of a quantum particle, e.g., a proton, by means of the Feynman Path-Integral formalism. In the simplest case, a classical system composed of a closed-ring polymer with adjacent replicas connected with harmonic springs is isomorphic to the quantum one. Some of the approximate approaches to deal with dynamical observables (e.g., Thermostatted Ring Polymer MD, TRPMD; or Partially Adiabatic Centroid MD, PACMD, both used in the above-presented work by Marsalek et al. [376]) provide access to the time-evolved properties by propagating the centroid motion in real time. These approximate approaches may suffer from issues, like the curvature problem in centroid MD [379], which is particularly severe at low temperatures [380], or ZPE-leakage [381] and spurious resonances occurring in TRPMD [381,382]. Recently, Althorpe and co-workers have proposed an alternative approach called Quasi-Centroid MD (QCMD) which circumvents these limitations [380,383]. More rigorous Path-Integral MD (PIMD) approaches have also been developed, yet at a clear computational cost [384,385,386,387].

**Figure 15:**
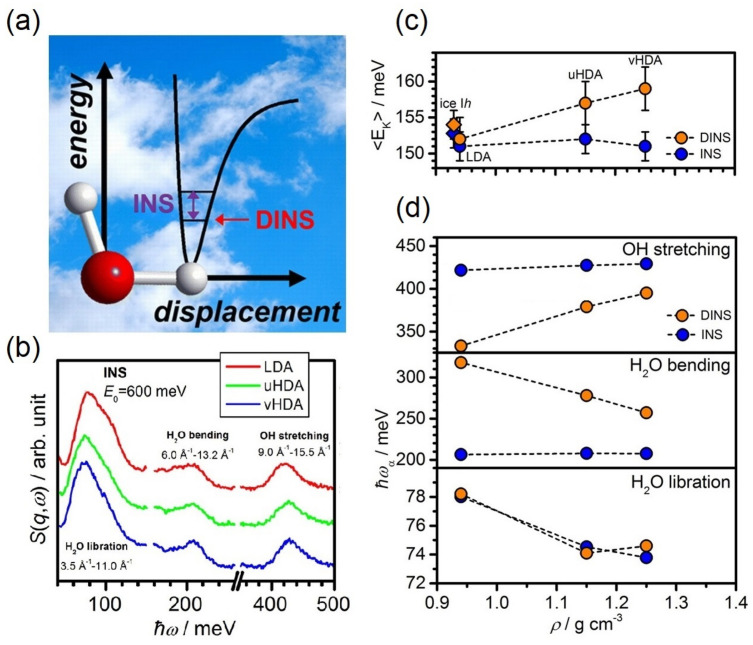
(**a**) Pictorial representation of the anharmonic potential of the OH-stretch mode of H_2_O. The vibrational ZPE measured with NCS (DINS), as well as the fundamental 0 → 1 transition probed with INS, are highlighted. (**b**) Experimental INS spectra of different forms of amorphous ice (LDA, red; unannealed HDA, green; and vHDA, blue) measured at T = 80 K on MARI. The spectra were obtained after averaging over the indicated *Q*-range. (**c**) Mean kinetic energies (<E_K_>) from NCS (orange) and corresponding values obtained from INS using the harmonic model discussed in Ref. [399] (blue). (**d**) Energies of the OH-stretch, bend, and librational modes of H_2_O, obtained from INS (blue), as well as the corresponding values derived from NCS (orange). Adapted with permission from Ref. [391]. Copyright (2021) American Chemical Society.

When NQEs are at the center of attention, experimental access to nuclear mean kinetic energies *<E_K_>* and associated momentum distributions *n(p)* via NCS becomes particularly relevant. As shown in Figure 14, local bending and stretching modes can be considered as descriptors of the interplay between NQEs arising from ZPE and the strength of H-bonds [389]. As discussed by Senesi et al. [390], the above-mentioned observables probed with NCS allow for a direct characterization of H-bonded systems. For example, it is now well-established that the strengthening the O–H covalent bonds broadens the associated proton momentum width probed with NCS. The tail of the *n(p)* distribution can be associated with the momentum along the O-H bond direction, and a large momentum width reflects a tightness of the proton binding, providing information about the strength and anharmonicity of the associated local vibrations. Upon increasing the H-bond strength, the effective proton potential will soften and, consequently, the proton *<E_K_>* and the O-H stretch frequency will decrease [390]. These considerations have been used by Parmentier et al. [391] to follow the evolution of the H-bond in amorphous forms of ice as a function of their density, clearly illustrating the complementarity between INS and NCS to characterize these systems (see Figure 15). Within a simplified (one-dimensional) model of the potential along the OH stretch, the cartoon in this Figure illustrates the primary differences between INS and NCS. INS probes the 0→ 1 excitation of a given mode from its vibrational ground state (an energy difference), whereas NCS measures directly the total mean kinetic energy associated with ZPEs, by operating within the so-called Impulse Approximation (IA). The bottom panel in Figure 15 displays the INS spectra measured on MARI (ISIS, UK) for three types of amorphous ice (LDA, uHDA, and vHDA, listed in order of increasing density). Three distinct bands in each INS spectrum can be associated to the water libration, bending, and OH-stretch modes. Similarly, NCS can provide information on directional mean kinetic energies, which can be further associated with these three types of vibrations and the associated ZPE contributions, for further comparison with INS (see Reference [391] for an extensive discussion). For a perfectly harmonic system, the value of the ZPE measured by both techniques would be, in principle, indistinguishable. The combination of both INS and NCS experiments allows, therefore, a further quantification of the degree of anharmonicity. The authors have focused on the OH-stretch mode dominating the total mean kinetic energy of the proton and, in so doing, have determined effective anharmonic constants for each amorphous phase. The mean kinetic energies were found to increase with increasing density, indicating the weakening of H-bonds, as well as a trend toward a steeper and more harmonic vibrational manifold.

The work by Parmentier et al. [391] highlights the power of NCS to retrieve ground-state kinetic energies without any underlying assumptions, which can be used as an accurate reference for testing and developing advanced theoretical approaches to account for NQEs. Since path-integral simulations converge slowly with the number of replicas used describe quantum behavior, the cost associated with their use alongside AIMD continues to be prohibitively high in most situations. The above-discussed use of neural networks to approximate the potentials with ab initio accuracy paves the way for obtaining fully converged quantum simulations [392], as well as opens new opportunities for making these approaches more widespread. This trend is also corroborated by considerable improvements in stochastic thermostatting [393,394], new methods to accelerate these simulations [395,396], as well as advanced tools now available to the wider community [397,398]. Such developments are providing synergies across experiment and theory, to understand and quantify NQEs at an unprecedented level of detail [399,400,401,402,403].

**Figure 16:**
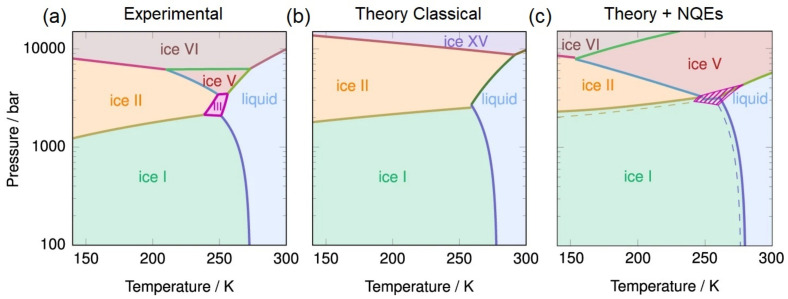
Experimental (**a**) and computational (**b**,**c**) phase diagrams of H_2_O in the range T = 50–300 K and *p* = 0.02–1 GPa. The computational phase diagrams have been obtained with classical (**b**) and PIMD simulations (**c**) using a vdW-corrected hybrid-DFT (revPBE0-D3). See Ref. [407] for more details. Adapted with permission from Ref. [407]

All of these advances can be illustrated further by returning to the first question posed in this Section, relating to how many structures of ice remain to be discovered. One way to tackle this challenge is the the calculation of accurate water phase diagrams from first-principles. Recent work demonstrates that progress to date looks promising, in spite of the difficulties associated with deficiencies in commonly used DFT approximations, as well as the need to incorporate NQEs from the outset—e.g., GGA predicts that ice sinks in water [363,404]. The SCAN functional is providing promising results, as it gives the observed trend in density across liquid water and hexagonal ice [363]. Following the achievements with neural-network simulations of SCW discussed earlier in this Section, Zhang et al. have presented a model that reproduces accurately the potential energy surface of the SCAN approximation of water, from low temperatures and pressures to about 2400 K and 50 GPa [405]. Using this potential and classical MD simulations, agreement with experimental data is generally satisfactory, correctly predicting the fluid, molecular and ionic phases of water, and almost all stable ice polymorphs, both ordered and disordered. Ice III and ice XV remain notable exceptions to the above, as they are stable but are found metastable in the simulations [406]. This result is quite intriguing, considering what is known on the polymorphs of ice XV. Nonetheless, the work from Zhang et al. [406] seems to be the first attempt with proven success to describe water over such a wide range of thermodynamic conditions without the inclusion of NQEs. Using ML-FF trained at the hybrid-functional level, Cheng et al. [405] have demonstrated that taking into account NQEs, anharmonic fluctuations, and proton disorder, it is possible to describe the structural and thermodynamic properties of liquid water, as well as hexagonal (Ih) and cubic (Ic) ice [405], in excellent agreement with experiments. This work also reports reliable estimates of the melting points of light and heavy water from first-principles, and has established that the increased stability of ice Ih over ice Ic stems from NQEs. Following this strategy, Reinhardt and Cheng have recently reported the phase diagram of water over the range T = 150–300 K and *p* = 0.01–1 GPa at the hybrid-DFT level, including NQEs [407]. These exciting results are shown in Figure 16. Starting from liquid water and a comprehensive set of fifty hypothetical ice structures waiting to be discovered, they show that none of them is thermodynamically stable, at least on a computer. This result suggests the completeness of the experimental phase diagram of water within these thermodynamic conditions. Figure 16 clearly evinces the importance of accounting for NQEs in the description of the phase diagram: forms III and V would not exist; and the proton-ordered ice-XV phase would be more stable than its disordered analogue ice VI [407]. In addition, the inclusion of NQEs improves the quantitative agreement with experimental data, including the not-so-trivial calculation of chemical potentials, particularly for ice III. This powerful computational framework is already being applied beyond water, with proven success in simulations of other systems discussed in this review. Such is the case of the application of the SCAN functional to the study of OHPs, including: its validation against accurate many-body theories (Random Phase Approximation, RPA) [408]; its superb performance when applied to the study of finite-temperature effects [409]; or in the description of their phase diagrams [410]. As recently demonstrated by Kapil et al. [411], the introduction of accelerated simulation techniques with the use of ML-FF are now becoming affordable to tackle phenomena, such as the aforementioned loading of guest molecules into extremely flexible MOF scaffolds. Furthermore, it opens new opportunities to address challenges across soft matter and polymer science. These range from dynamical phenomena and glassy behavior exhibiting significant NQEs [368,412,413,414], macromolecular thermophysics [415], isotope effects on polymer crystallization [416,417] or the chemistry of polymerization, including extreme conditions [418].

## 5. Outlook

The past decade has witnessed very significant advances in neutron spectroscopy, and this work has made an attempt to provide a timely overview of the use of this tool in the study of complex materials, with a focus on those of a soft and polymeric nature. Our choices in the selection of the areas of research and scientific drivers described herein have been unavoidably colored by our personal preferences, appreciation, and knowledge of an otherwise vast and rapidly growing area of activity. The field of OHPs constitutes a good case-in-point, as it is by now abundantly clear that the degree of complexity at the atomic scale associated with these soft solids requires the insights brought forward by the technique, as highlighted in Section 2 above. In this regard, INS stands out as the method of choice to scrutinize their behavior, particularly if coupled to first-principles computational materials modeling. Similar remarks would equally apply to the study of confined soft matter and supramolecular frameworks, as covered in Section 3. In this case, progress has also required parallel developments in the requisite sample-environment equipment in order to carry out in-situ, *operando*, or high-pressure studies, now possible owing to order-of-magnitude improvements in measurement speeds. The proliferation in the number of experiments on MOFs and COFs in the past years summarized in Table 1 is testament to the above. At a fundamental level, these studies have taught us that both structure and function in these nanoporous media are inextricably linked to dynamics and motion. Once more, a robust interpretation of INS data is predicated upon the use of computational modeling, and much progress has been achieved in parallel in the reliable description of these rather large systems with the requisite level of accuracy. Last but not least, going back to water in Section 4 has been intentional for several reasons. The level of complexity exhibited by this seemingly simple molecular system across its phase diagram continues to baffle us. State-of-the-art INS experiments as those illustrated in Figure 11 represent a novel means of resolving ongoing (and often heated) controversies on this pivotal system, simply thought unthinkable not so long ago. Moreover, the role played by NQEs can now be probed across the phase diagram with NCS, and these experiments are also motivating further and exciting theoretical developments. The spectacular shifts in phase boundaries shown in Figure 16 as NQEs are switched ‘on’ and ‘off’ are a tantalizing reminder of how much remains to be explored, taking water as a starting point in an otherwise long and fruitful journey into other classes of materials.

It is certainly tempting (perhaps foolhardy) to end with a few words on the road ahead. As qualitative scientific advances generally follow developments in our tools and methods of inquiry, the advent of next-generation neutron facilities, like the European Spallation Source, will certainly take us to a whole new level over the next few years. As such, we very much hope that this review provides a spring board for the community to capitalize from these in new and original ways.

## Data Availability

Not applicable.
